# Supplementary material for: Design, Synthesis and Biological Evaluation of Novel Triazole N-acylhydrazone Hybrids for Alzheimer’s Disease
Source: Molecules. 2020 Jul 10;25(14):3165. doi: 10.3390/molecules25143165 (PMC7397262; doi:10.3390/molecules25143165)
Supplement: Supplementary file 1 [file molecules-25-03165-s001.pdf]

Design, Synthesis and Biological Evaluation of Novel Triazole *N*-acylhydrazone Hybrids for Alzheimer's Disease

Matheus de Freitas Silva[1*], Ellen Tardelli Lima[1], Letizia Proccoli[2], Newton G. Castro[3], Marcos Jorge R. Guimarães[3], Fernanda M. R. da Silva[3], Nathalia Fonseca Nadur[4], Luciana Luiz de Azevedo[4], Arthur Eugen Kümmerle[4], Isabella Alvim Guedes[5], Laurent Emmanuel Dardenne[5], Vanessa Silva Gontijo[1], Andrea Tarozzi[2*]; and Claudio Viegas Jr[1*]

1. Laboratory of Research in Medicinal Chemistry (PeQuiM), Federal University of Alfenas, Jovino Fernandes Sales Avenue, 2600, Alfenas/MG, 37130-000, Brazil; defreitassilva.matheus@gmail.com (M.d.F.S.); ellentflima17@gmail.com (E.T.L); vanessagontijo@yahoo.com.br (V.S.G.); cvjviegas@gmail.com (C.V.J.)
2. Department for Life Quality Studies, Alma Mater Studiorum-University of Bologna, Corso d'Augusto 237, 47921 Rimini, Italy; letizia.pruccoli2@unibo.it (L.P.); andrea.tarozzi@unibo.it (A.T.)
3. Laboratory of Molecular Pharmacology, Federal University of Rio de Janeiro, Avenida Carlos Chagas Filho, 373, Rio de Janeiro/RJ, 21941-590, Brazil; ngcastro@icb.ufrj.br (N.G.C.); mj.jorge93@gmail.com (M.J.R.G.); dasilvafmr@gmail.com (F.M.R.d.S.)
4. Laboratory of molecular Diversity and Medicinal Chesmistry (LaDMol-QM), Federal Rural University of Rio de Janeiro – UFRRJ, BR-465, Km 7 Seropédica-Rio de Janeiro; nathaliafn18@gmail.com (N.F.N.); lucianaluizazevedo@gmail.com (L.L.d.A.); akummerle@yahoo.com.br (A.E.K.)
5. Grupo de Modelagem Molecular em Sistemas Biológicos (GMMSB), National Laboratory for Scientific Computing - LNCC, Avenida Getúlio Vargas, 333, Petrópolis - RJ, Brazil; isabella.alvimg@gmail.com (I.A.G.); dardenne@lncc.br (L.E.D.)

*Correspondence: defreitassilva.matheus@gmail.com

Received: date; Accepted: date; Published: date

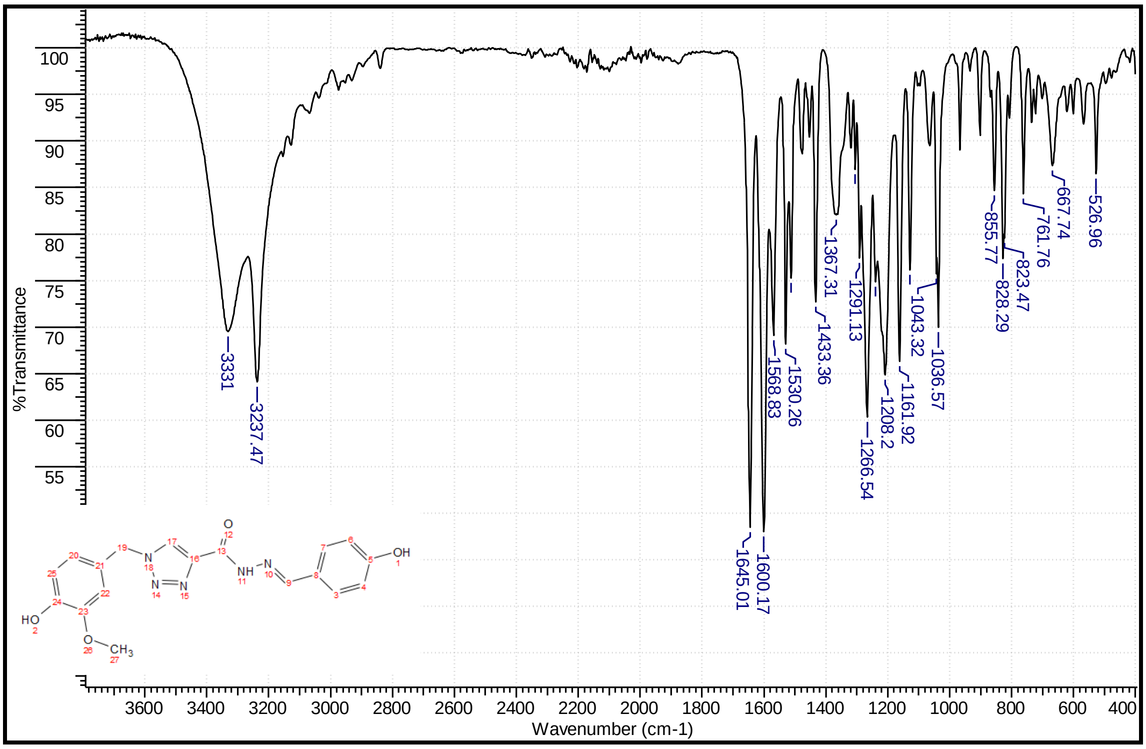

Figure S 1. Absorption spectra in the infrared region of (E)-1-(4-hydroxy-3-methoxybenzyl)-N'-(4-hydroxybenzylidene)-1H-1,2,3-triazole-4-carbohydrazide (3a).

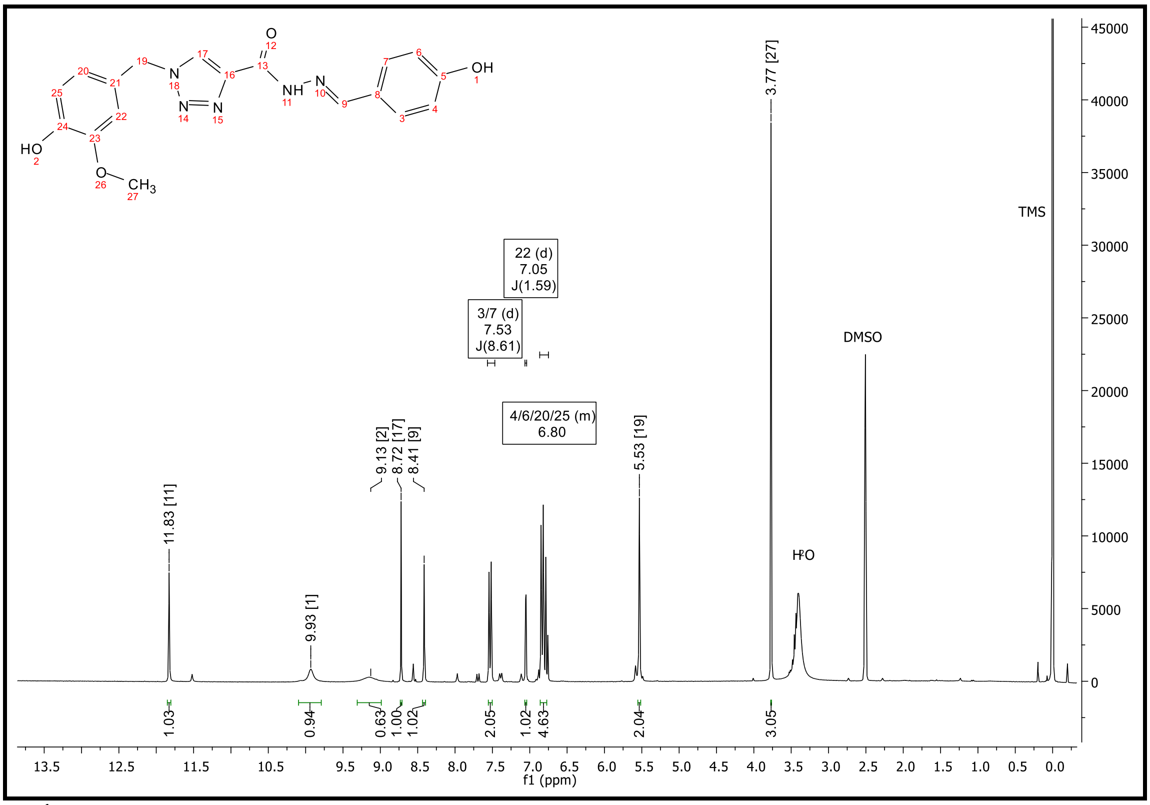

Figure S 2 ^{1}H NMR spectra of (E)-1-(4-hydroxy-3-methoxybenzyl)-N'-(4-hydroxybenzylidene)-1H-1,2,3-triazole-4-carbohydrazide (3a).

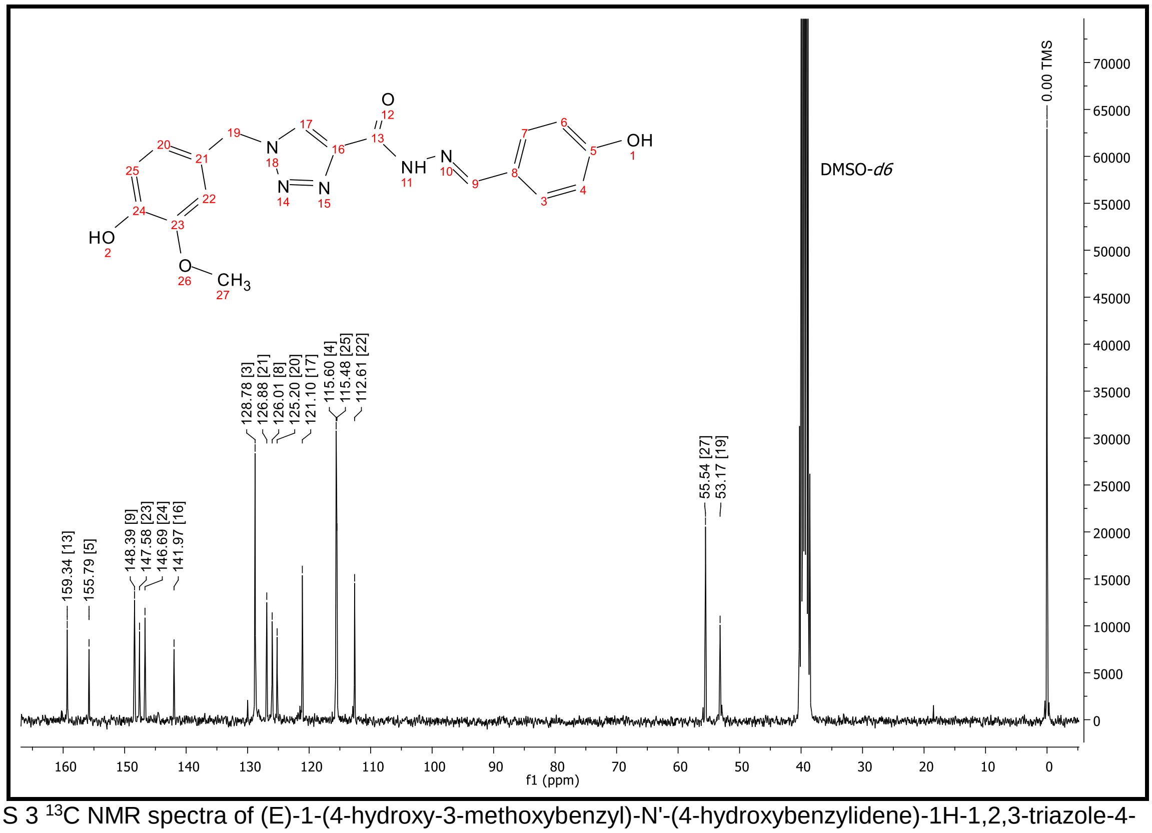

Figure S 3 ^{13}C NMR spectra of (E)-1-(4-hydroxy-3-methoxybenzyl)-N'-(4-hydroxybenzylidene)-1H-1,2,3-triazole-4-carbohydrazide (3a).

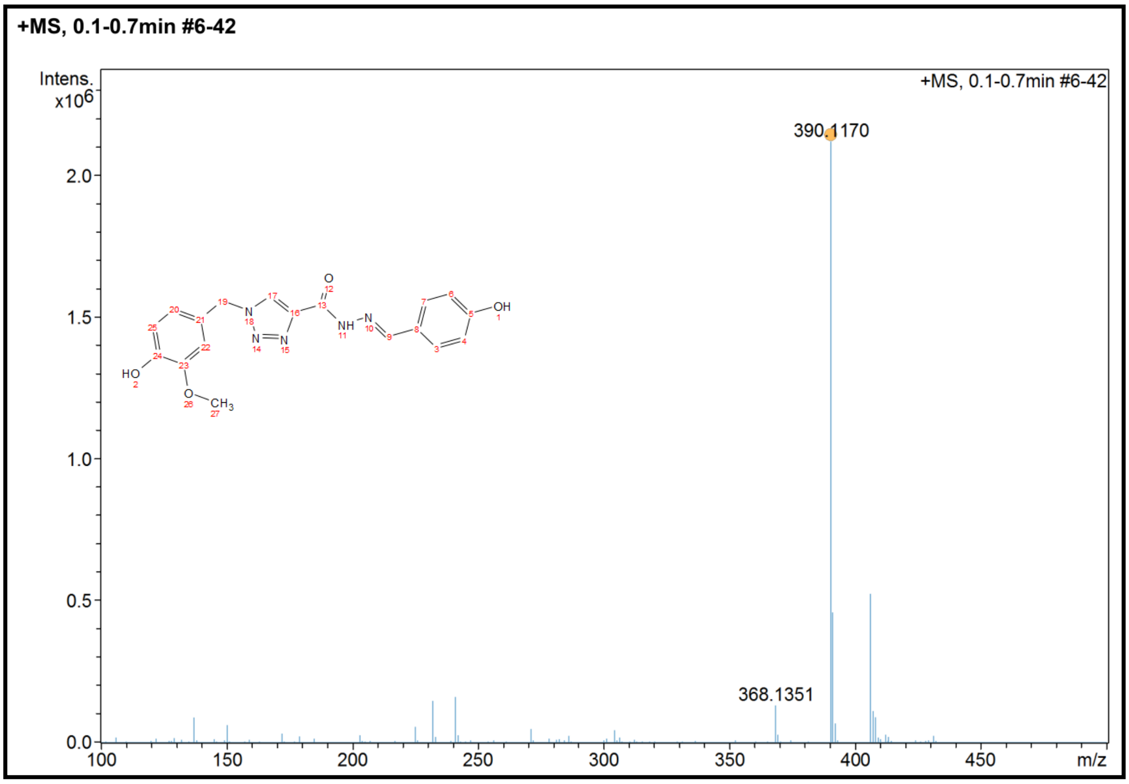

Figure S 4 . HR-MS spectra of (E)-1-(4-hydroxy-3-methoxybenzyl)-N'-(4-hydroxybenzylidene)-1H-1,2,3-triazole-4-carbohydrazide (3a).

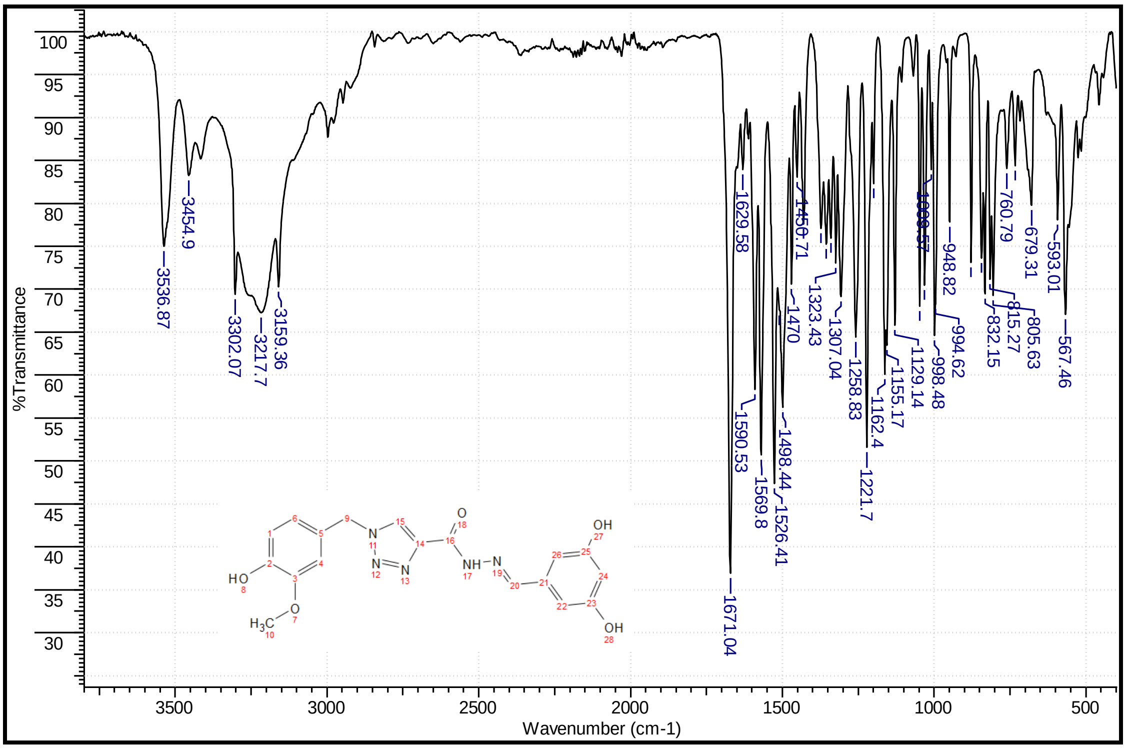

Figure S 5 Absorption spectra in the infrared region of (E)-N'-(3,5-dihydroxybenzylidene)-1-(4-hydroxy-3-methoxybenzyl)-1H-1,2,3-triazole-4-carbohydrazide (3b).

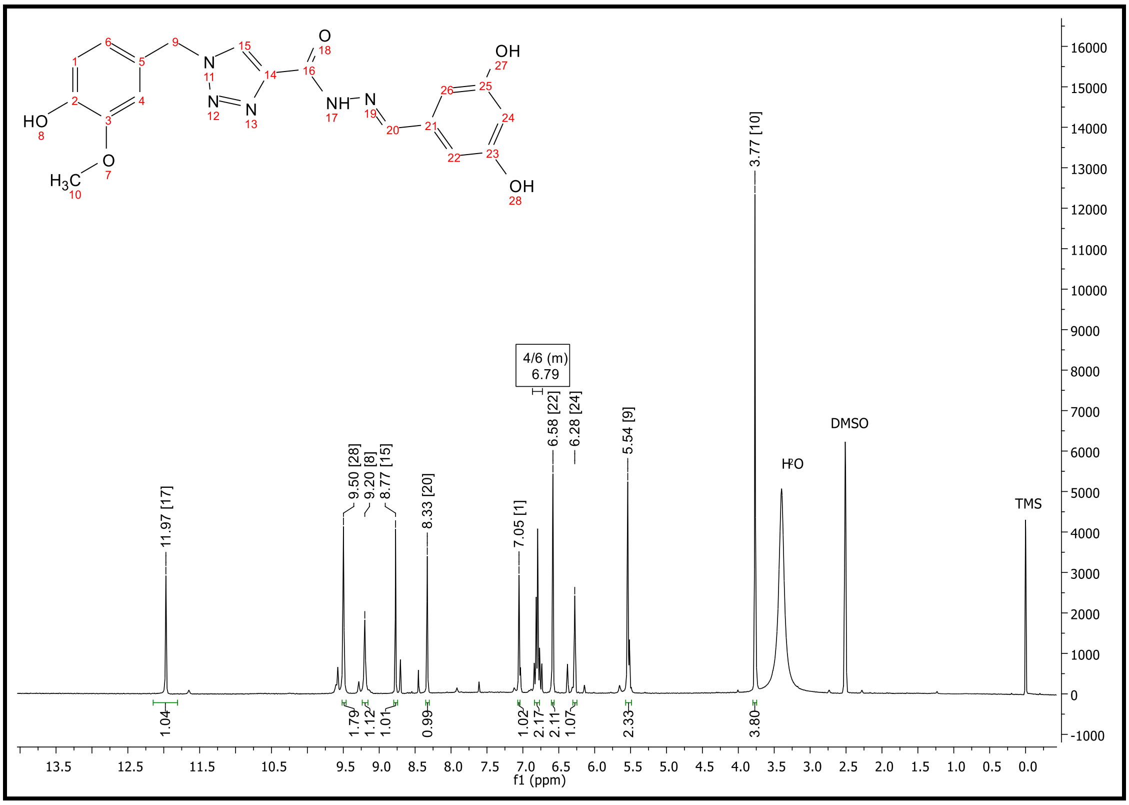

Figure S 6 1H NMR spectra of (E)-N'-(3,5-dihydroxybenzylidene)-1-(4-hydroxy-3-methoxybenzyl)-1H-1,2,3-triazole-4-carbohydrazide (3b).

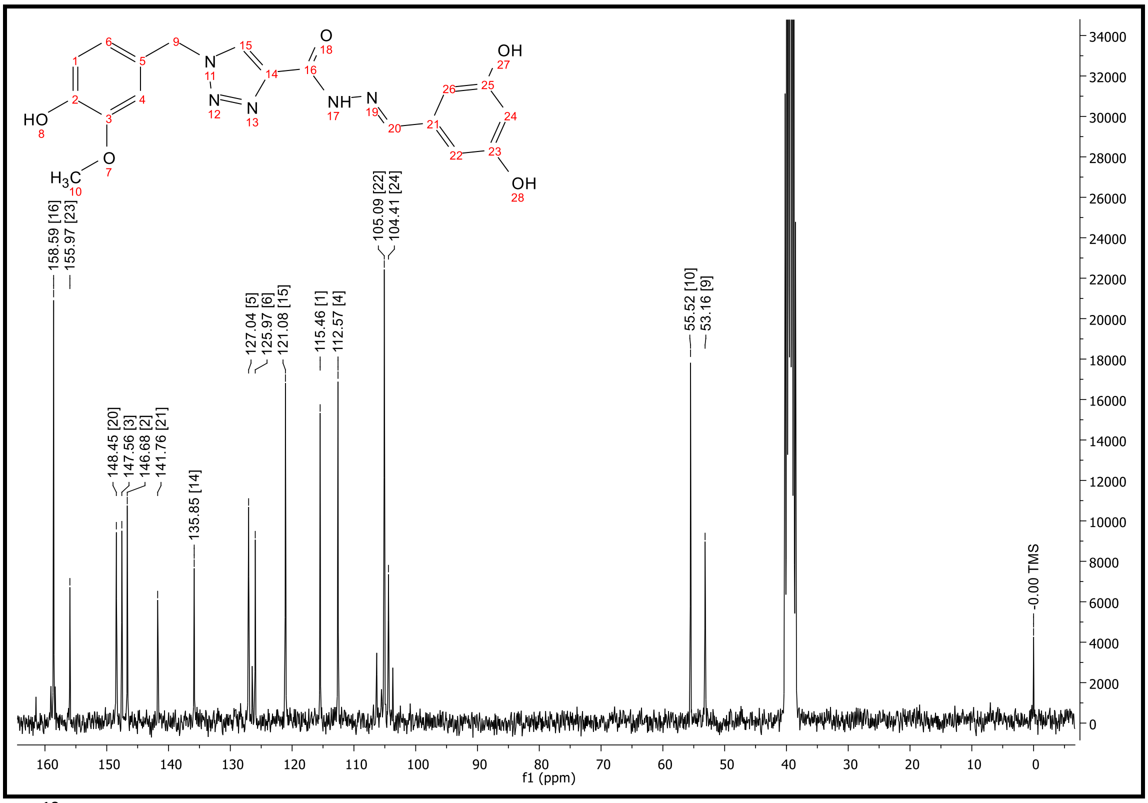

Figure S 7 ^{13}C NMR spectra of (E)-N'-(3,5-dihydroxybenzylidene)-1-(4-hydroxy-3-methoxybenzyl)-1H-1,2,3-triazole-4-carbohydrazide (**3b**).

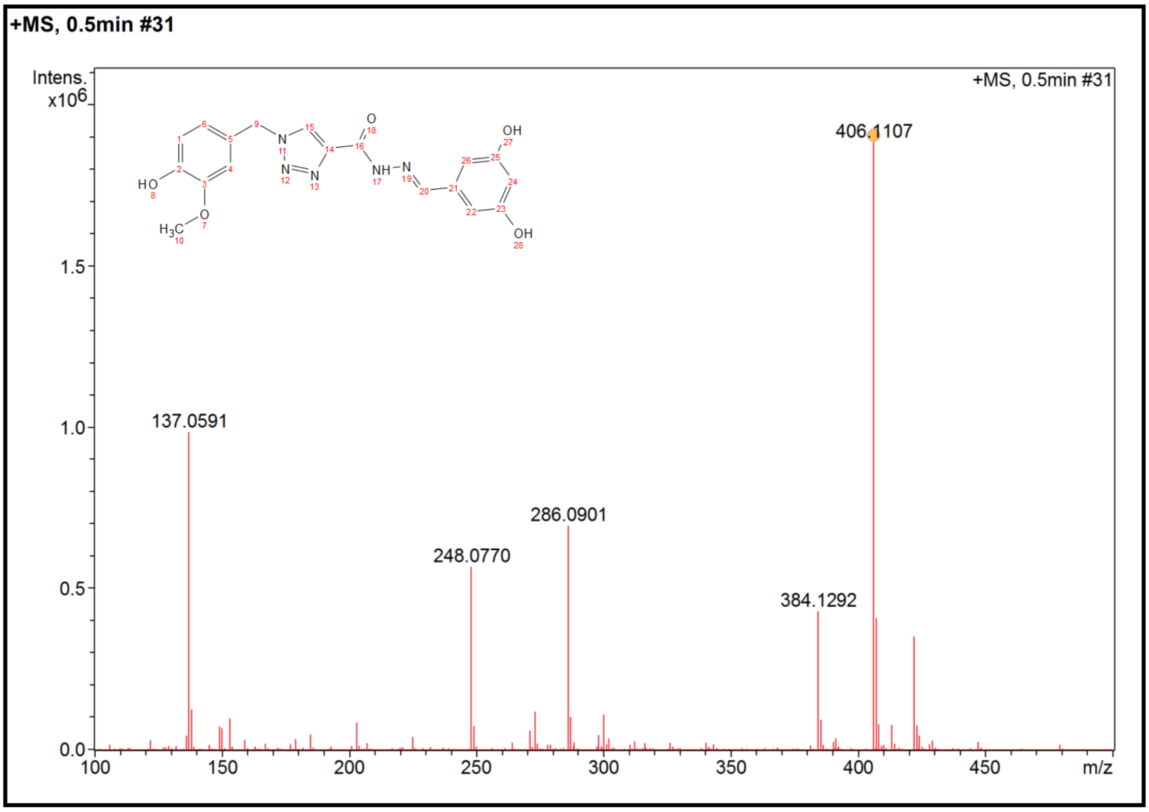

Figure S 8 HR-MS spectra of (E)-N'-(3,5-dihydroxybenzylidene)-1-(4-hydroxy-3-methoxybenzyl)-1H-1,2,3-triazole-4-carbohydrazide (**3b**).

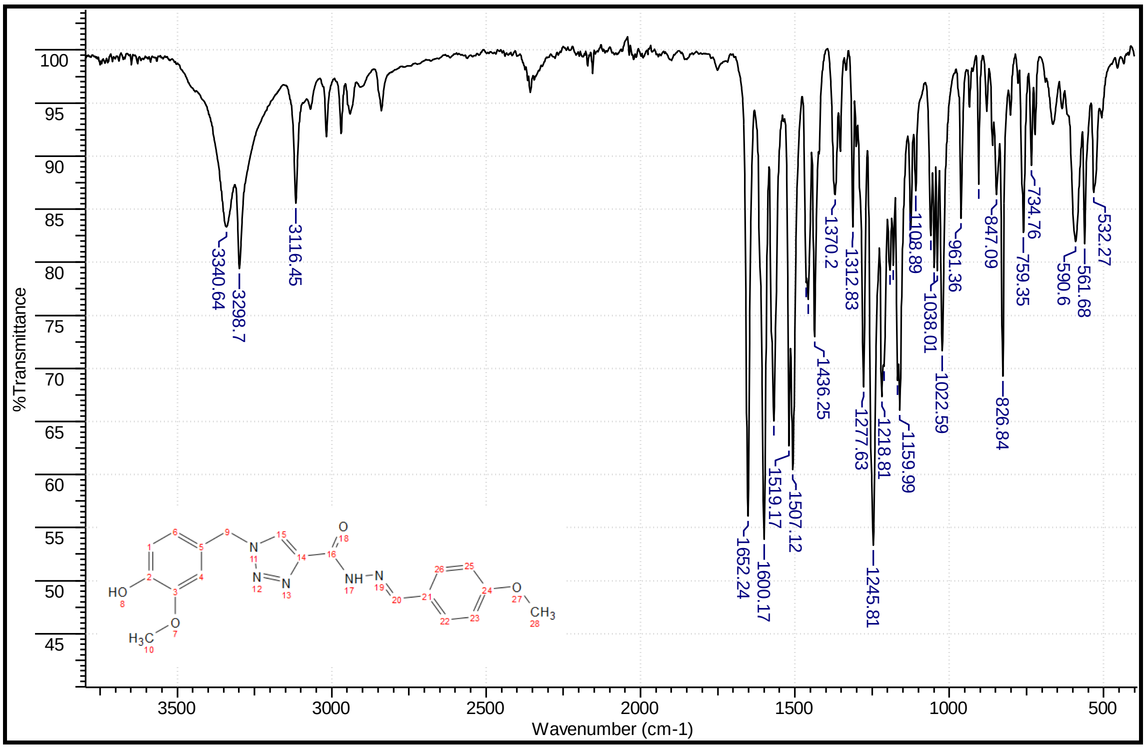

Figure S 9 Absorption spectra in the infrared region of (E)-1-(4-hydroxy-3-methoxybenzyl)-N'-(4-methoxybenzylidene)-1H-1,2,3-triazole-4-carbohydrazide (**3c**).

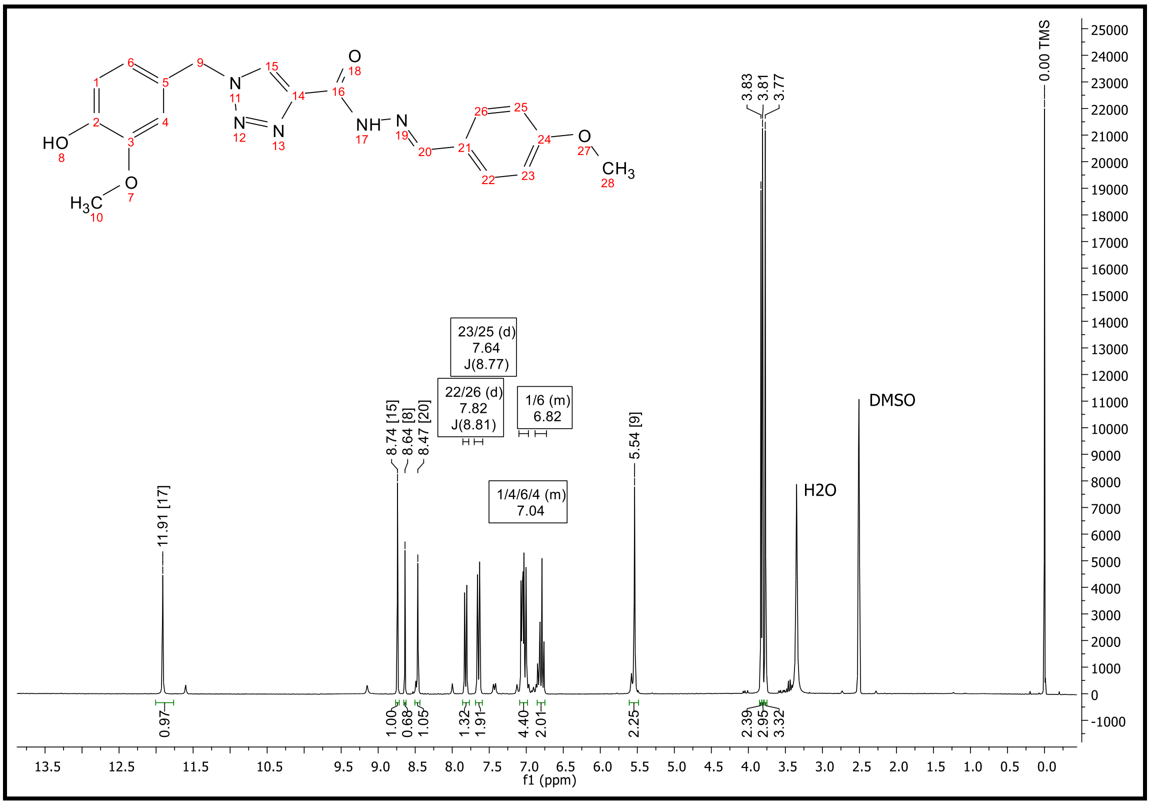

Figure S 1. 1H NMR spectra of (E)-1-(4-hydroxy-3-methoxybenzyl)-N'-(4-methoxybenzylidene)-1H-1,2,3-triazole-4-carbohydrazide (**3c**).

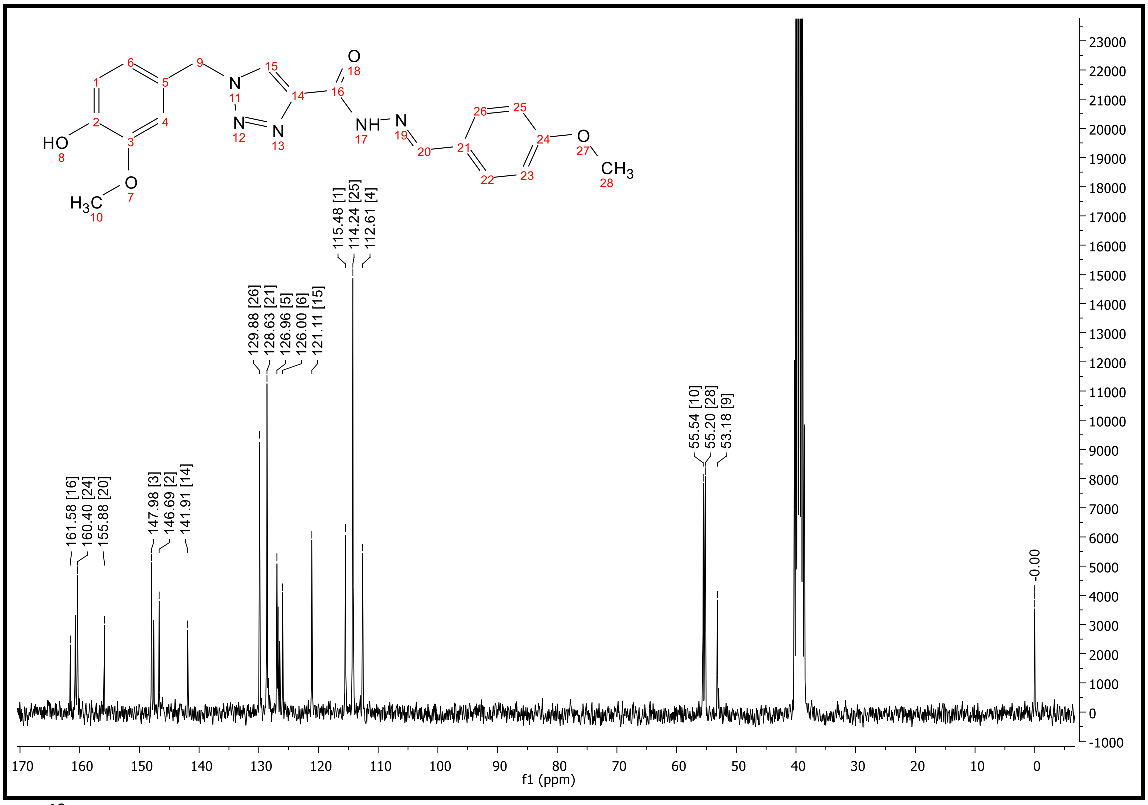

Figure S 2. ^{13}C NMR spectra of (E)-1-(4-hydroxy-3-methoxybenzyl)-N'-(4-methoxybenzylidene)-1H-1,2,3-triazole-4-carbohydrazide (**3c**).

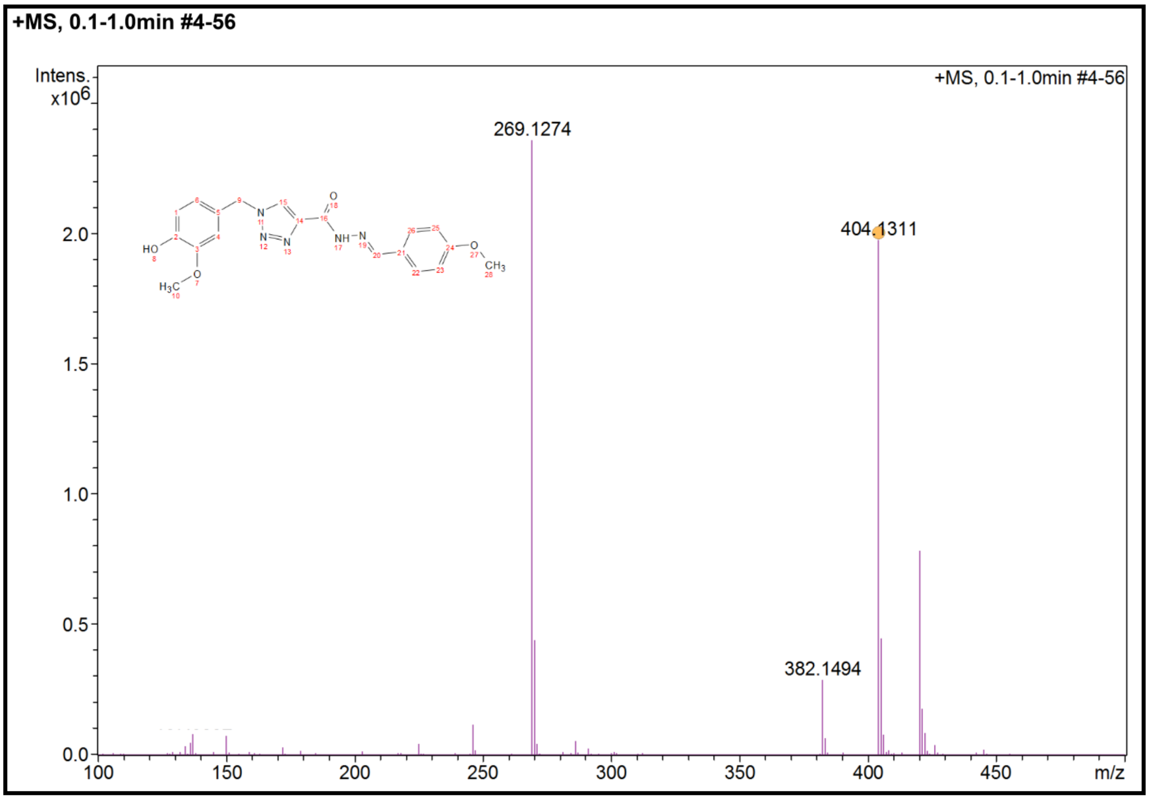

Figure S 3. HR-MS spectra of (E)-1-(4-hydroxy-3-methoxybenzyl)-N'-(4-methoxybenzylidene)-1H-1,2,3-triazole-4-carbohydrazide (**3c**).

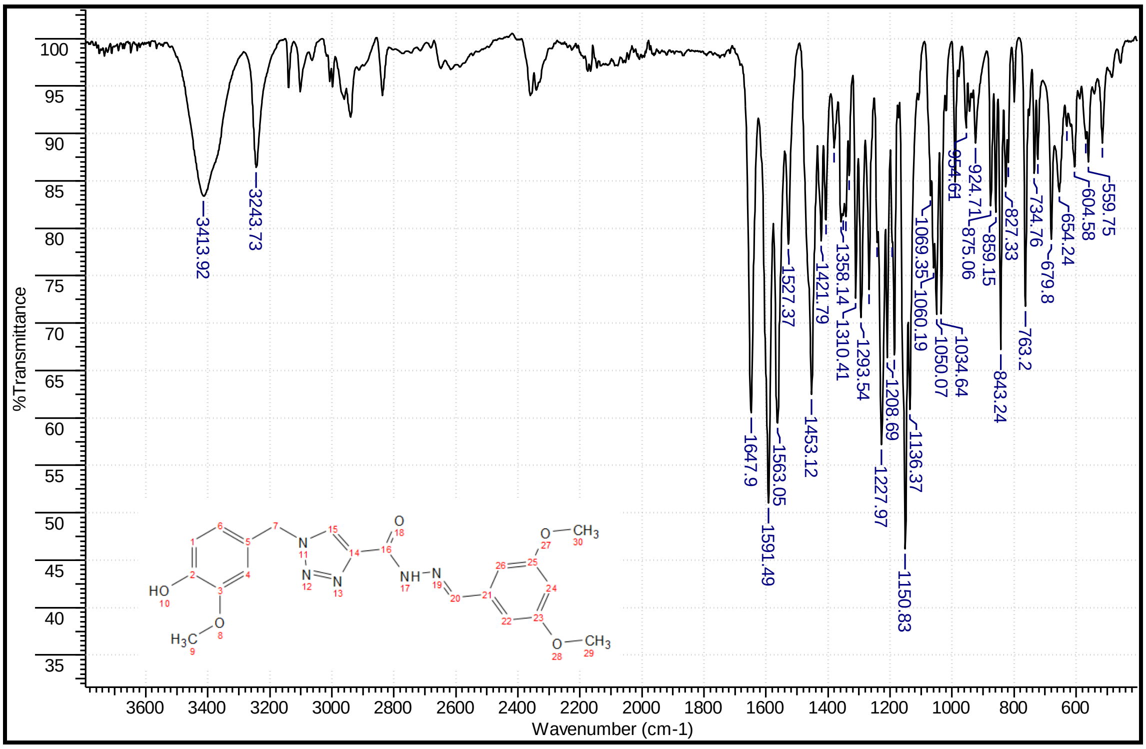

Figure S 4. Absorption spectra in the infrared region of (E)-N'-(3,5-dimethoxybenzylidene)-1-(4-hydroxy-3-methoxybenzyl)-1H-1,2,3-triazole-4-carbohydrazide (**3d**).

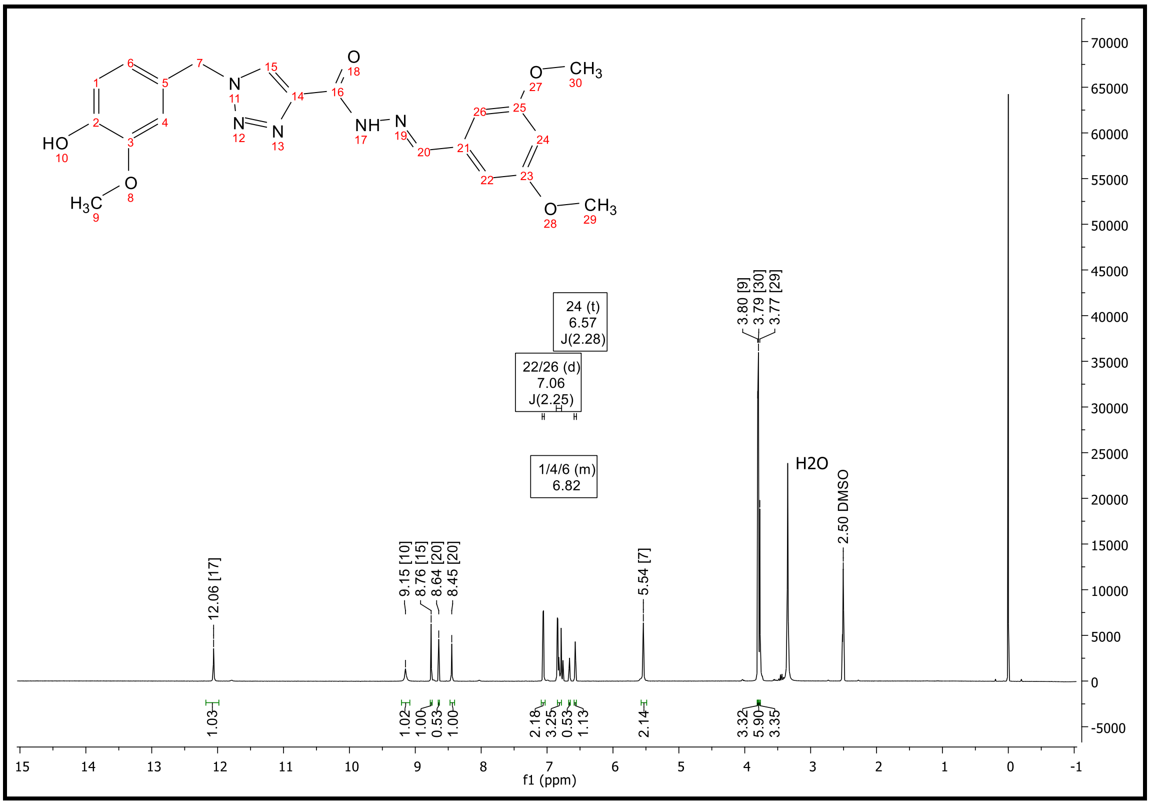

Figure S 5. 1H NMR spectra of (E)-N'-(3,5-dimethoxybenzylidene)-1-(4-hydroxy-3-methoxybenzyl)-1H-1,2,3-triazole-4-carbohydrazide (**3d**).

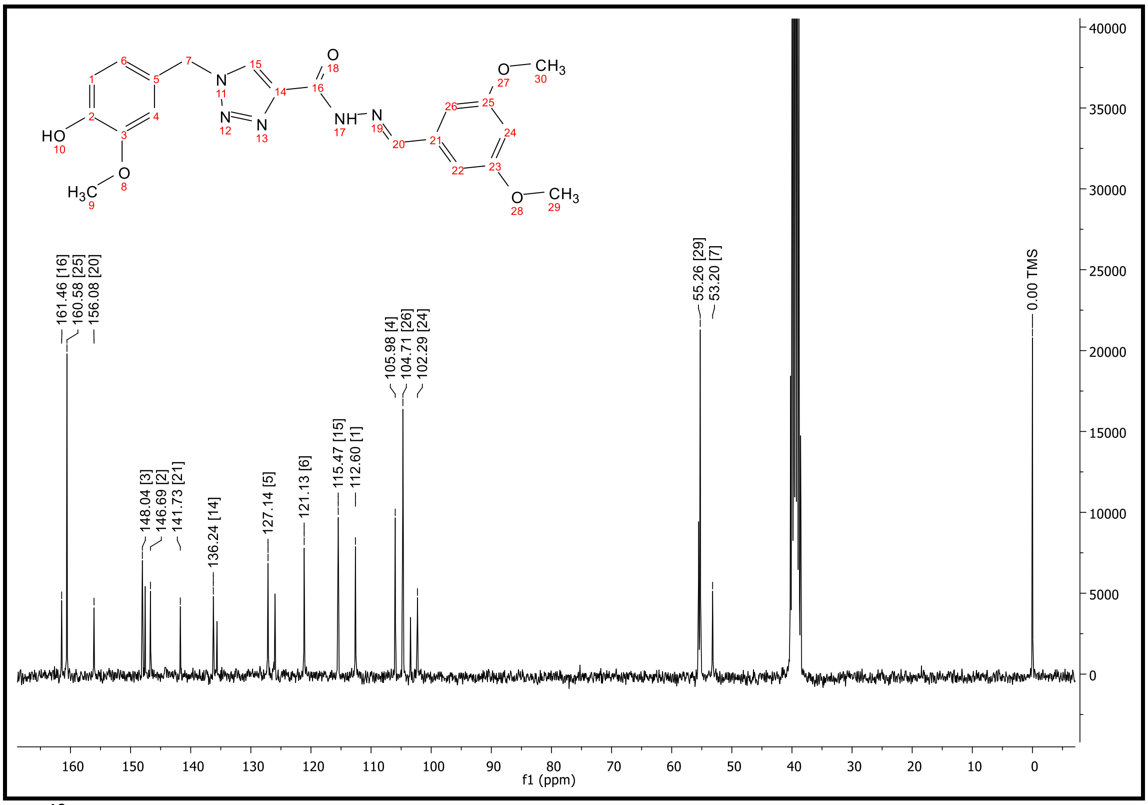

Figure S 6. ^{13}C NMR spectra of (E)-N'-(3,5-dimethoxybenzylidene)-1-(4-hydroxy-3-methoxybenzyl)-1H-1,2,3-triazole-4-carbohydrazide (**3d**).

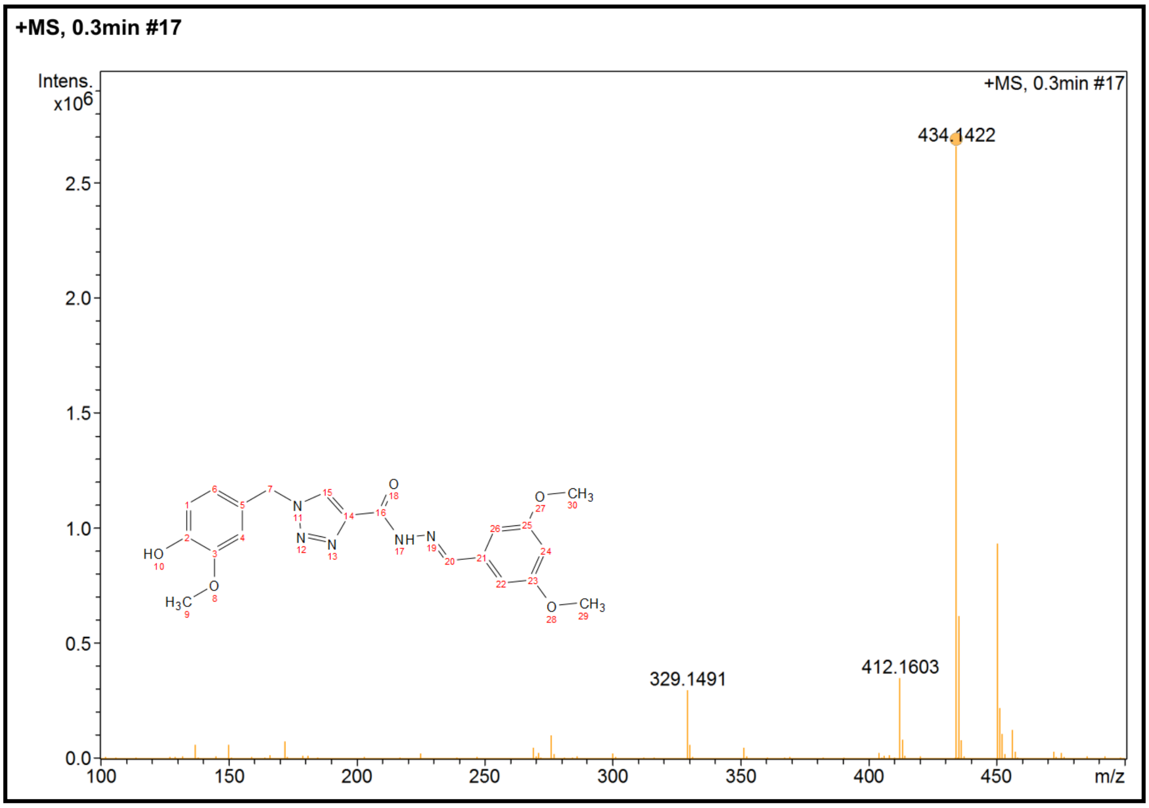

Figure S 7. HR-MS spectra of (E)-N'-(3,5-dimethoxybenzylidene)-1-(4-hydroxy-3-methoxybenzyl)-1H-1,2,3-triazole-4-carbohydrazide (**3d**).

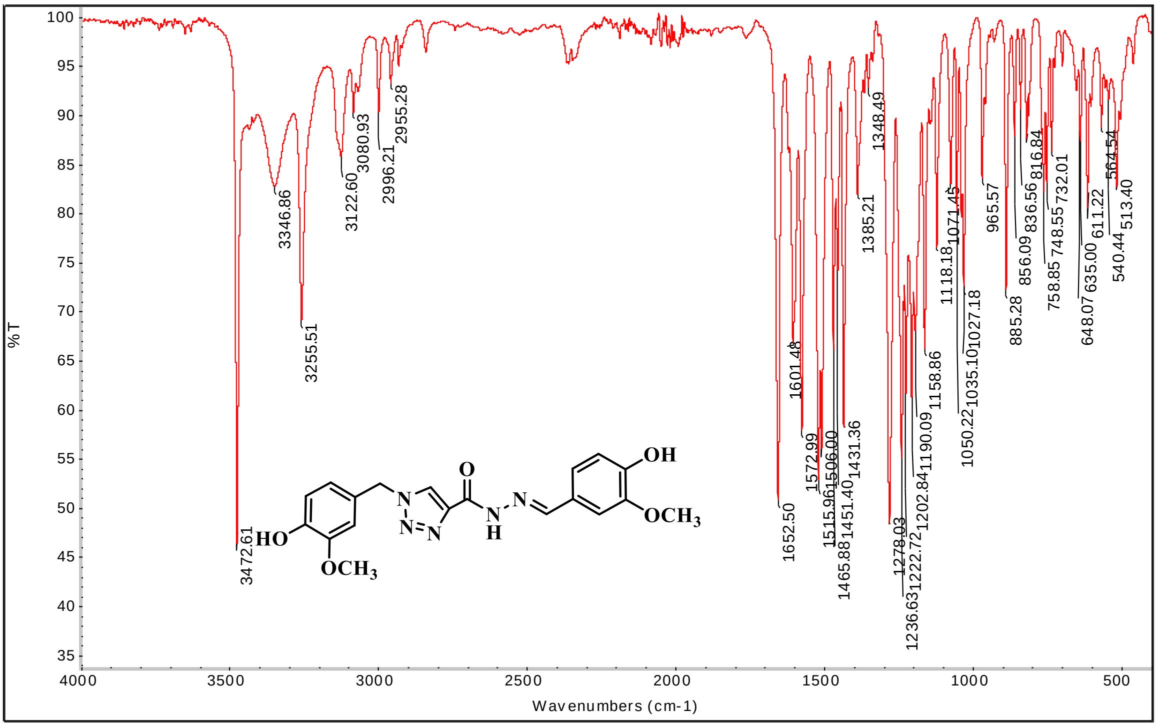

Figure S 8. Absorption spectra in the infrared region of (E)-1-(4-hydroxy-3-methoxybenzyl)-N'-(4-hydroxy-3-methoxybenzylidene)-1H-1,2,3-triazole-4-carbohydrazide (**3e**).

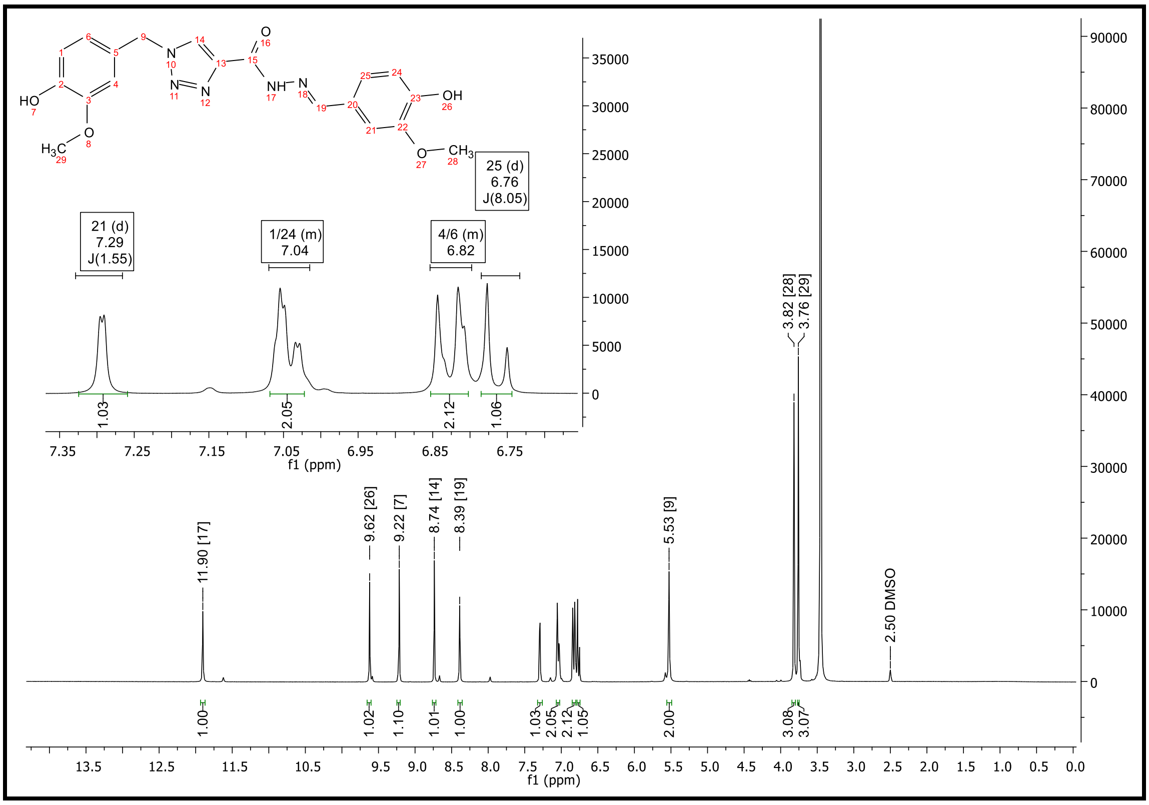

Figure S 9. 1H NMR spectra of (E)-1-(4-hydroxy-3-methoxybenzyl)-N'-(4-hydroxy-3-methoxybenzylidene)-1H-1,2,3-triazole-4-carbohydrazide (**3e**).

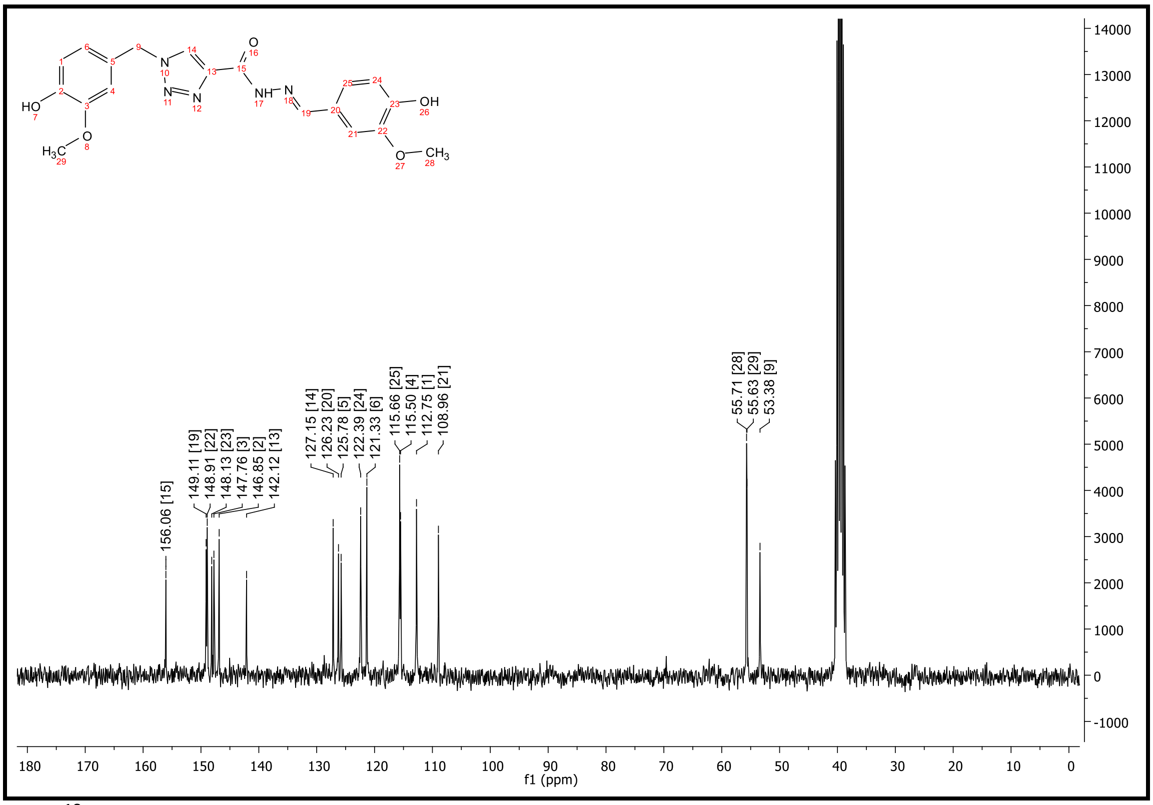

Figure S 10. ^{13}C NMR spectra of (E)-1-(4-hydroxy-3-methoxybenzyl)-N'-(4-hydroxy-3-methoxybenzylidene)-1H-1,2,3-triazole-4-carbohydrazide (**3e**).

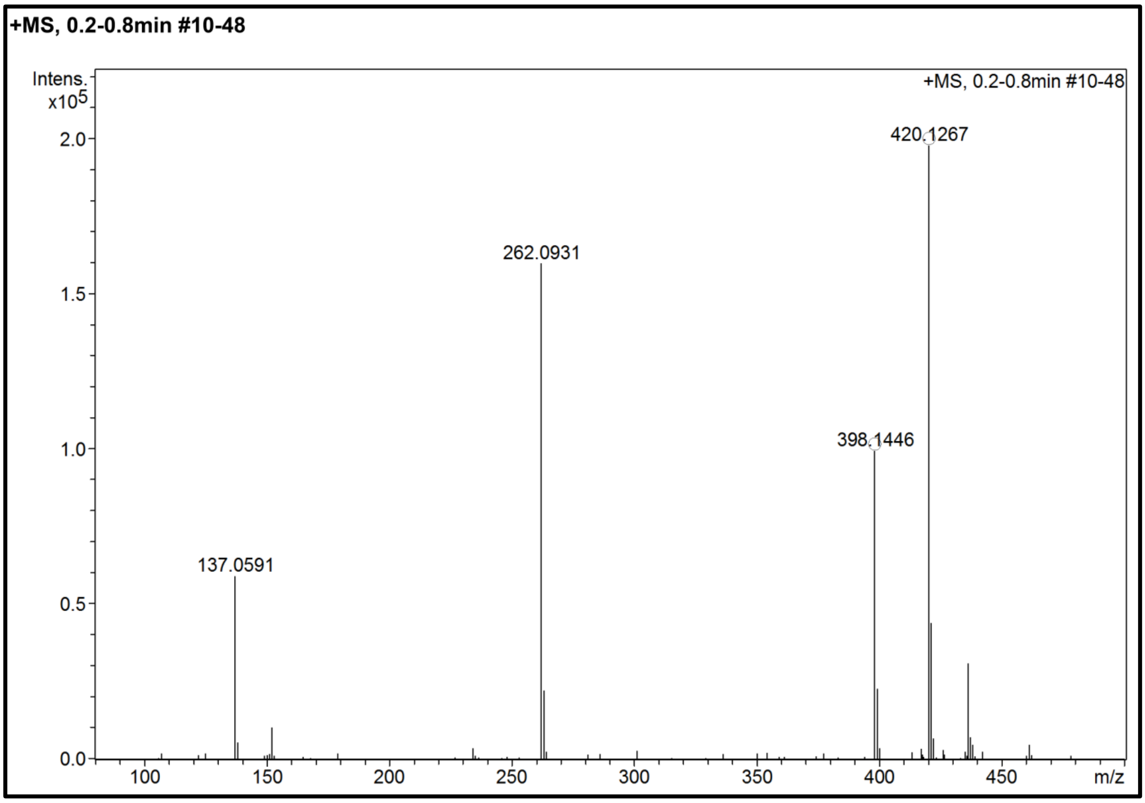

Figure S 11. HR-MS spectra of (E)-1-(4-hydroxy-3-methoxybenzyl)-N'-(4-hydroxy-3-methoxybenzylidene)-1H-1,2,3-triazole-4-carbohydrazide (**3e**).

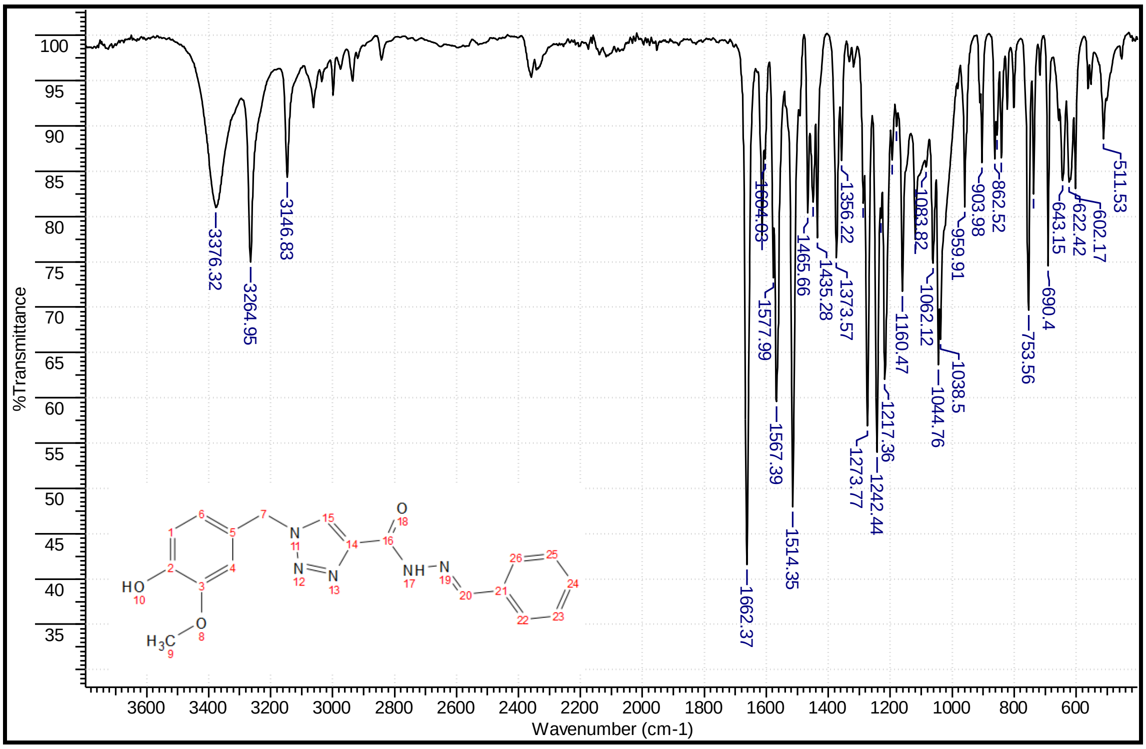

Figure S 12. Absorption spectra in the infrared region of (E)-N'-benzylidene-1-(4-hydroxy-3-methoxybenzyl)-1H-1,2,3-triazole-4-carbohydrazide (**3f**).

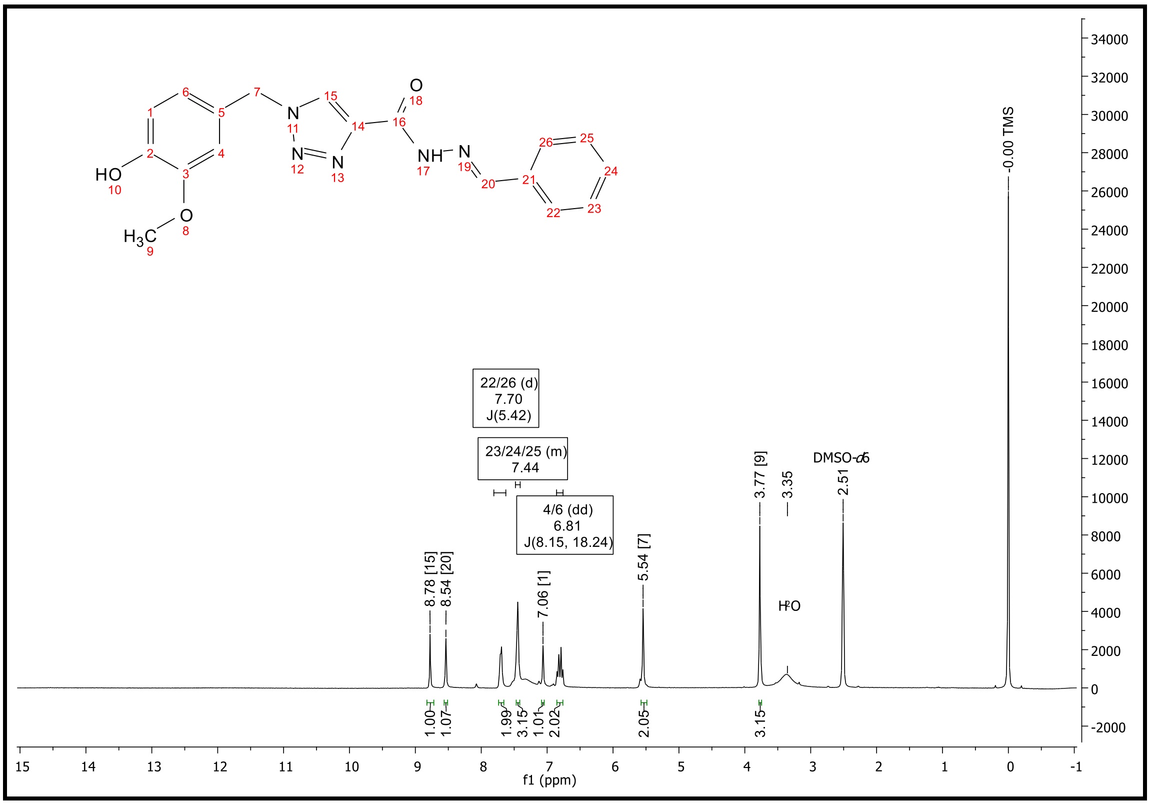

Figure S 13. 1H NMR spectra of (E)-N'-benzylidene-1-(4-hydroxy-3-methoxybenzyl)-1H-1,2,3-triazole-4-carbohydrazide (**3f**).

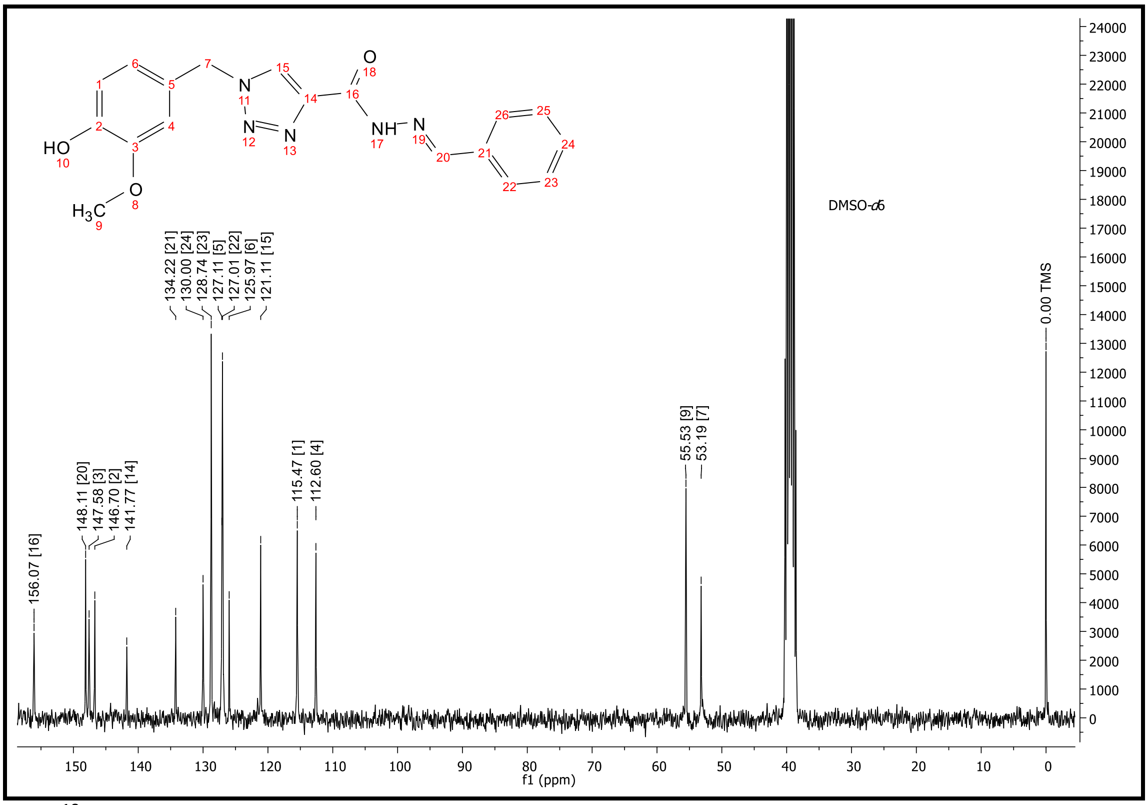

Figure S 14. ^{13}C NMR spectra of (E)-N'-benzylidene-1-(4-hydroxy-3-methoxybenzyl)-1H-1,2,3-triazole-4-carbohydrazide (**3f**).

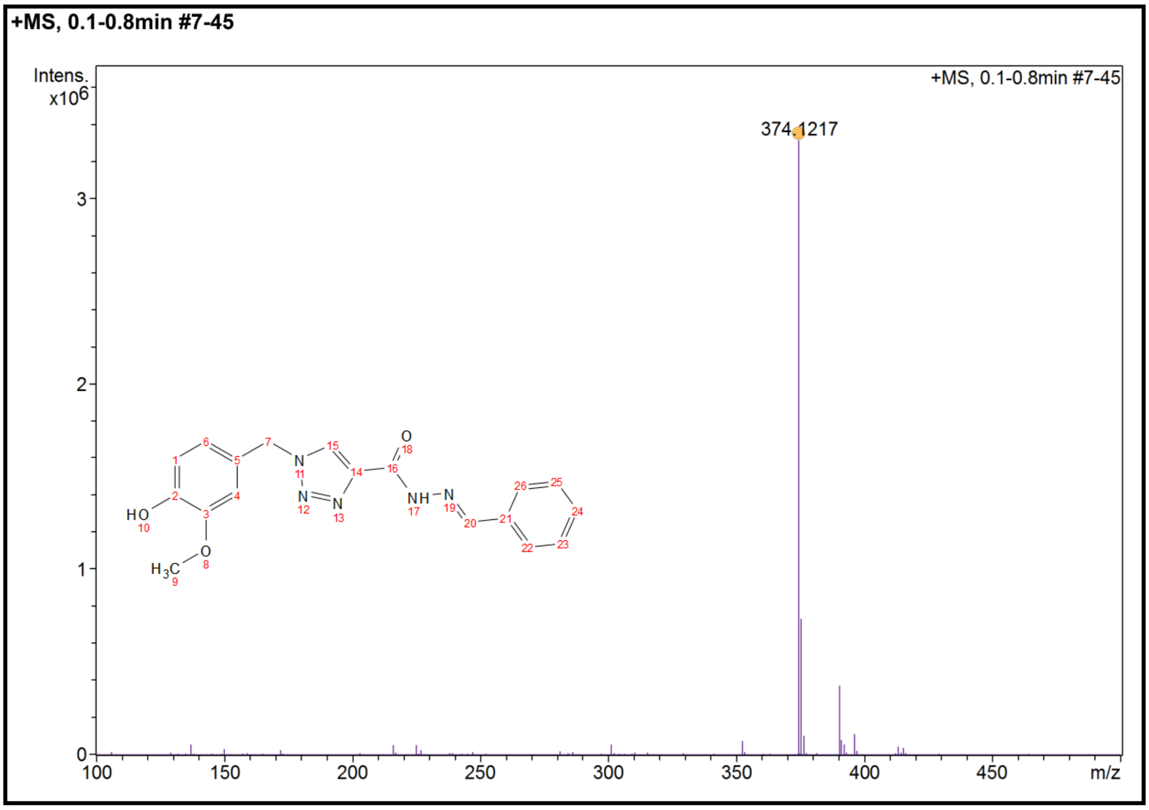

Figure S 15. HR-MS spectra of (E)-N'-benzylidene-1-(4-hydroxy-3-methoxybenzyl)-1H-1,2,3-triazole-4-carbohydrazide (**3f**).

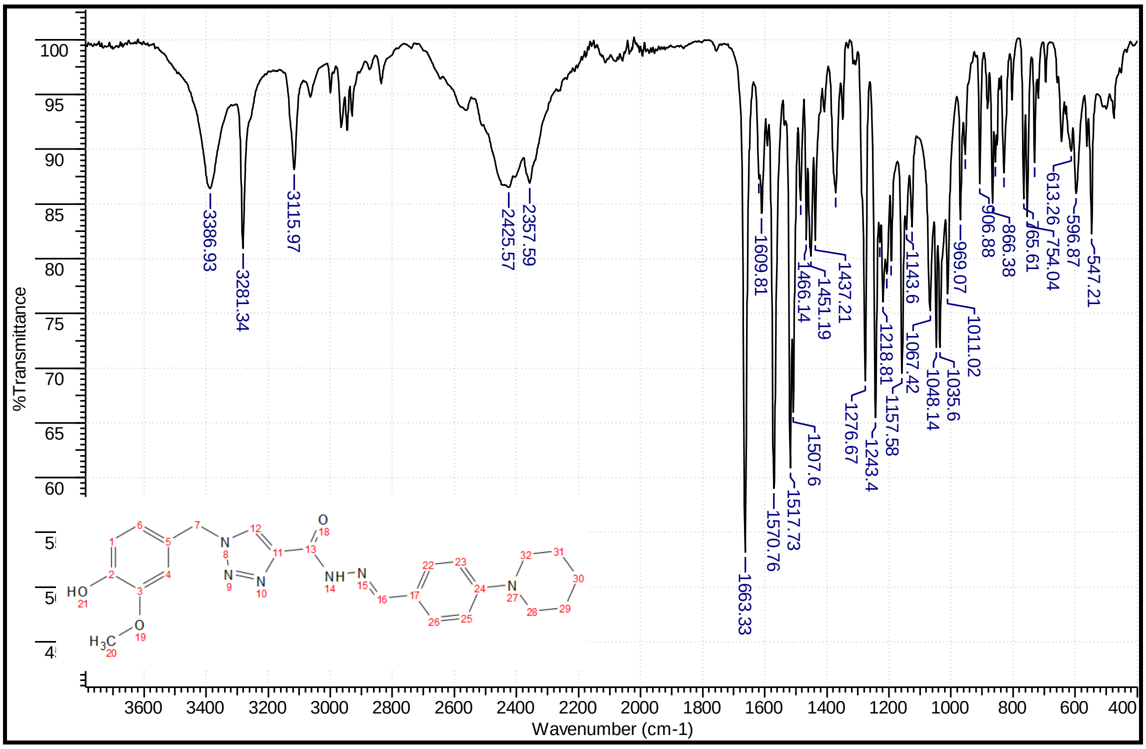

Figure S 16. Absorption spectra in the infrared region of (E)-1-(4-hydroxy-3-methoxybenzyl)-N'-(4-(piperidin-1-yl)benzylidene)-1H-1,2,3-triazole-4-carbohydrazide (**3g**).

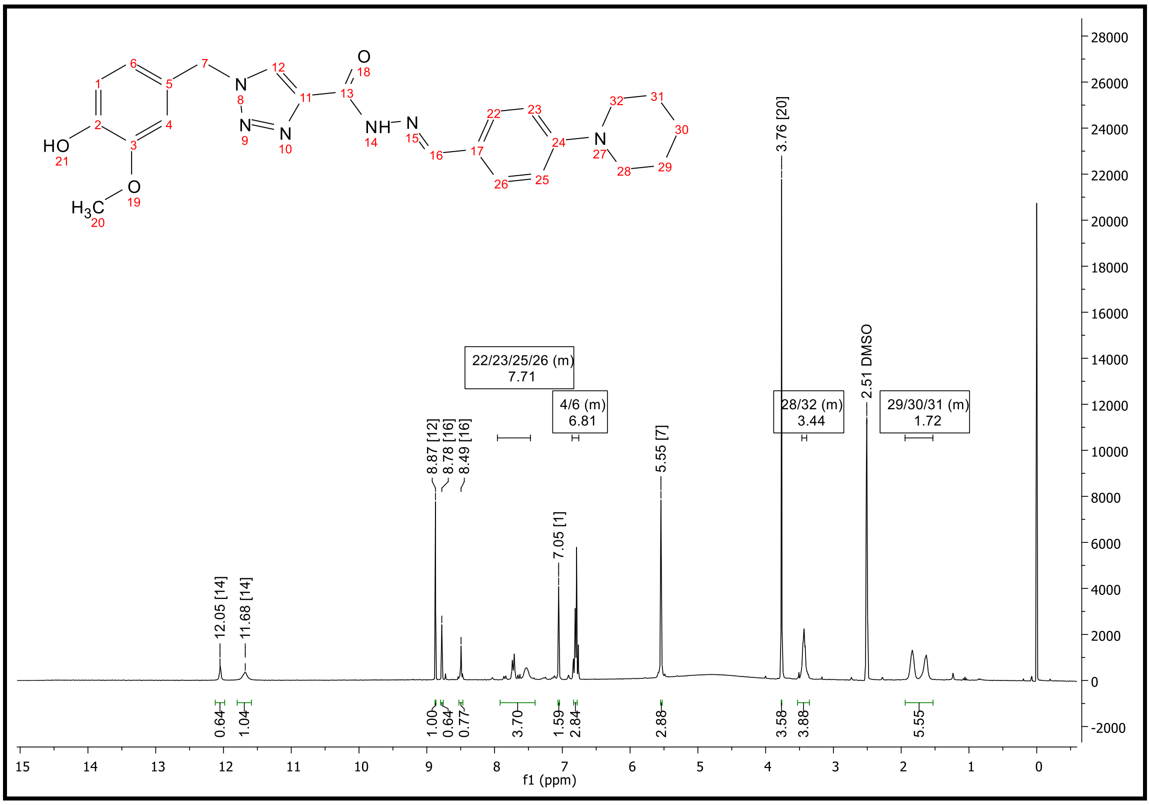

Figure S 17. 1H NMR spectra of (E)-1-(4-hydroxy-3-methoxybenzyl)-N'-(4-(piperidin-1-yl)benzylidene)-1H-1,2,3-triazole-4-carbohydrazide (**3g**).

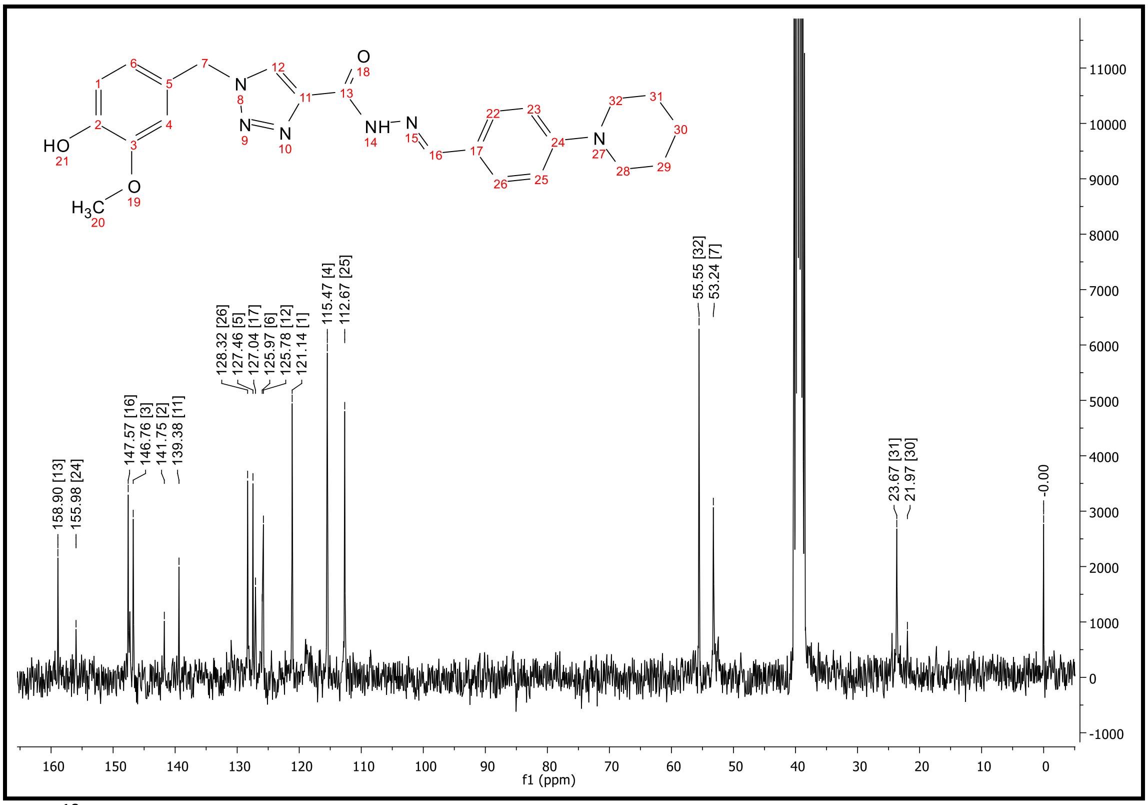

Figure S 18. ^{13}C NMR spectra of (E)-1-(4-hydroxy-3-methoxybenzyl)-N'-(4-(piperidin-1-yl)benzylidene)-1H-1,2,3-triazole-4-carbohydrazide (**3g**).

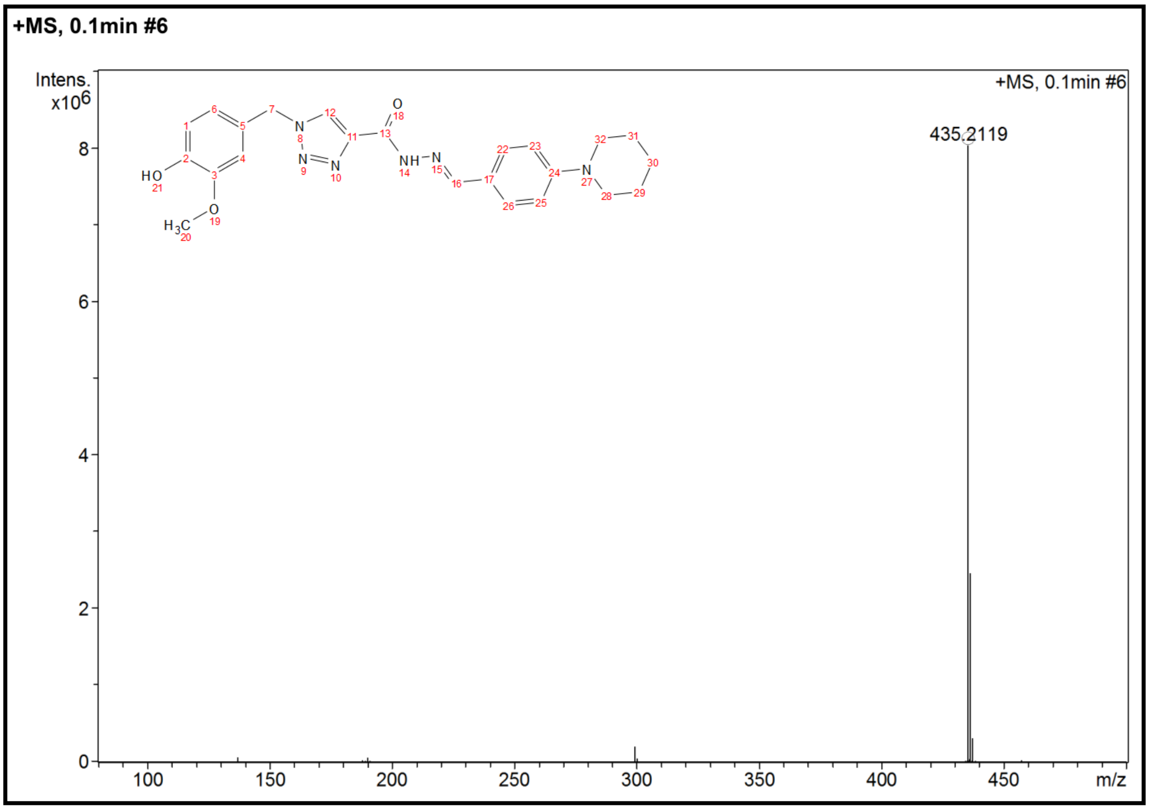

Figure S 19. HR-MS spectra of (E)-1-(4-hydroxy-3-methoxybenzyl)-N'-(4-(piperidin-1-yl)benzylidene)-1H-1,2,3-triazole-4-carbohydrazide (**3g**).

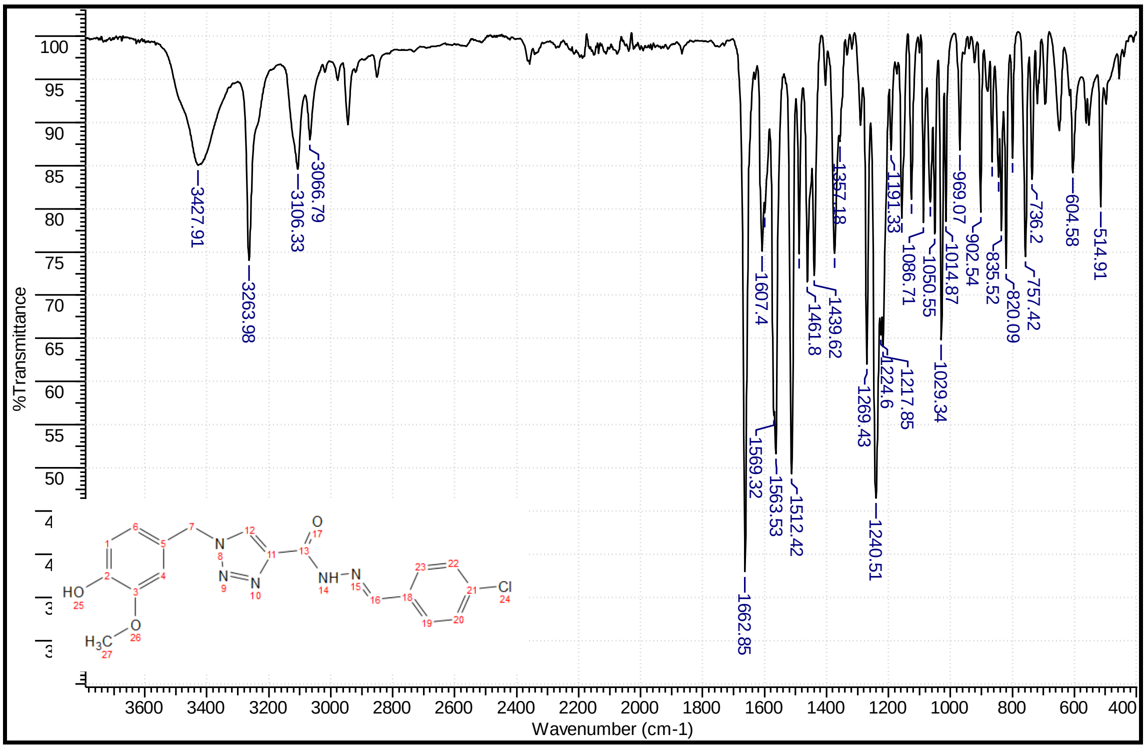

Figure S 20. Absorption spectra in the infrared region of (E)-N'-(4-chlorobenzylidene)-1-(4-hydroxy-3-methoxybenzyl)-1H-1,2,3-triazole-4-carbohydrazide (**3h**).

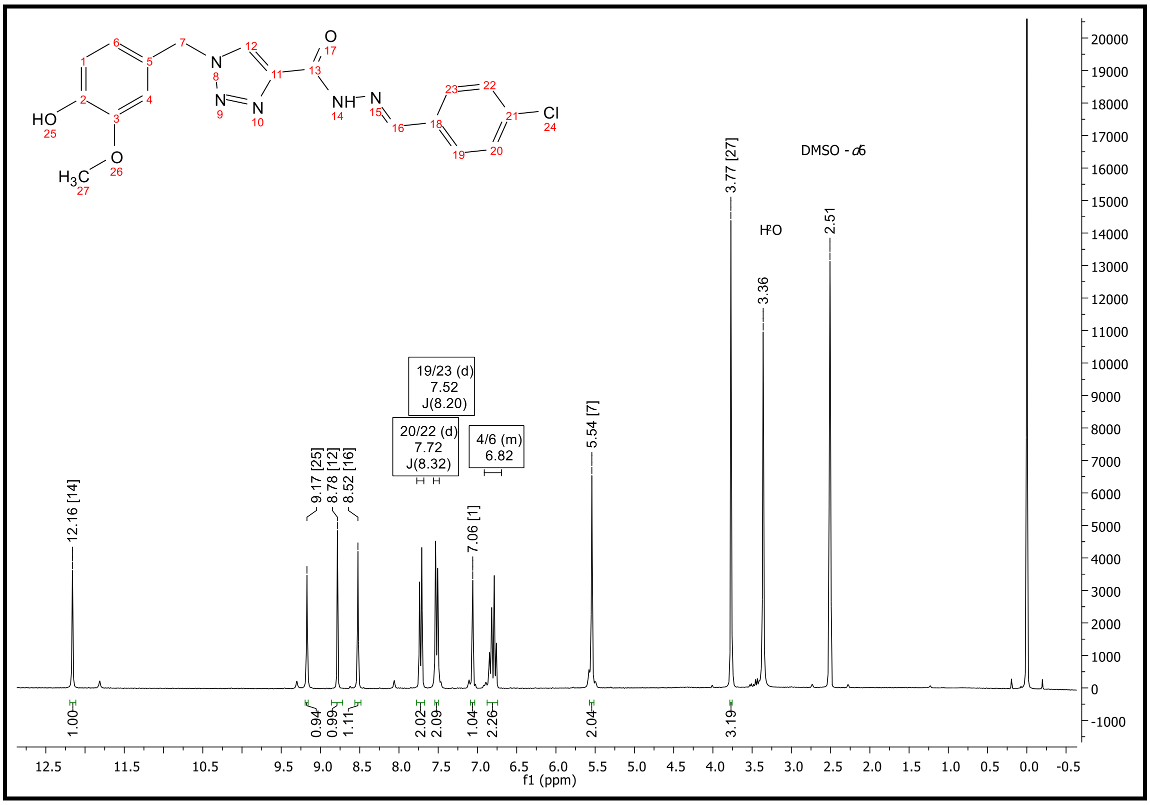

Figure S 21. 1H NMR spectra of (E)-N'-(4-chlorobenzylidene)-1-(4-hydroxy-3-methoxybenzyl)-1H-1,2,3-triazole-4-carbohydrazide (**3h**).

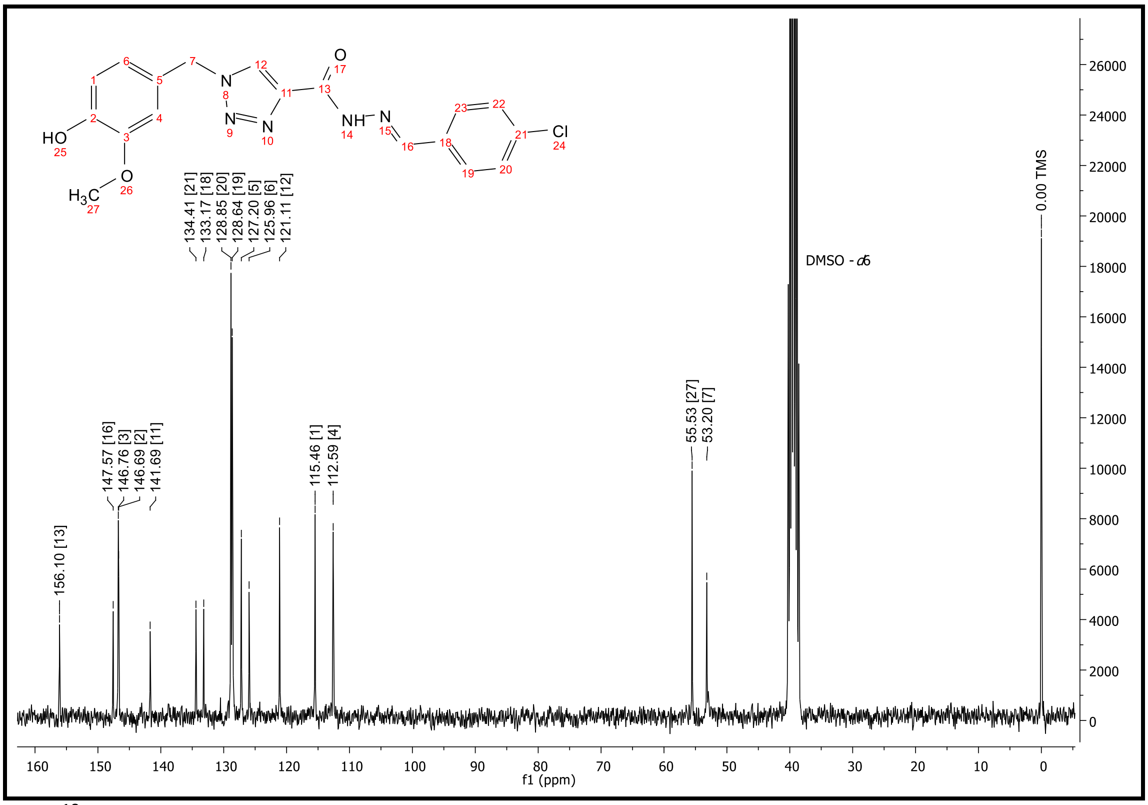

Figure S 22. ^{13}C NMR spectra of (E)-N'-(4-chlorobenzylidene)-1-(4-hydroxy-3-methoxybenzyl)-1H-1,2,3-triazole-4-carbohydrazide (**3h**).

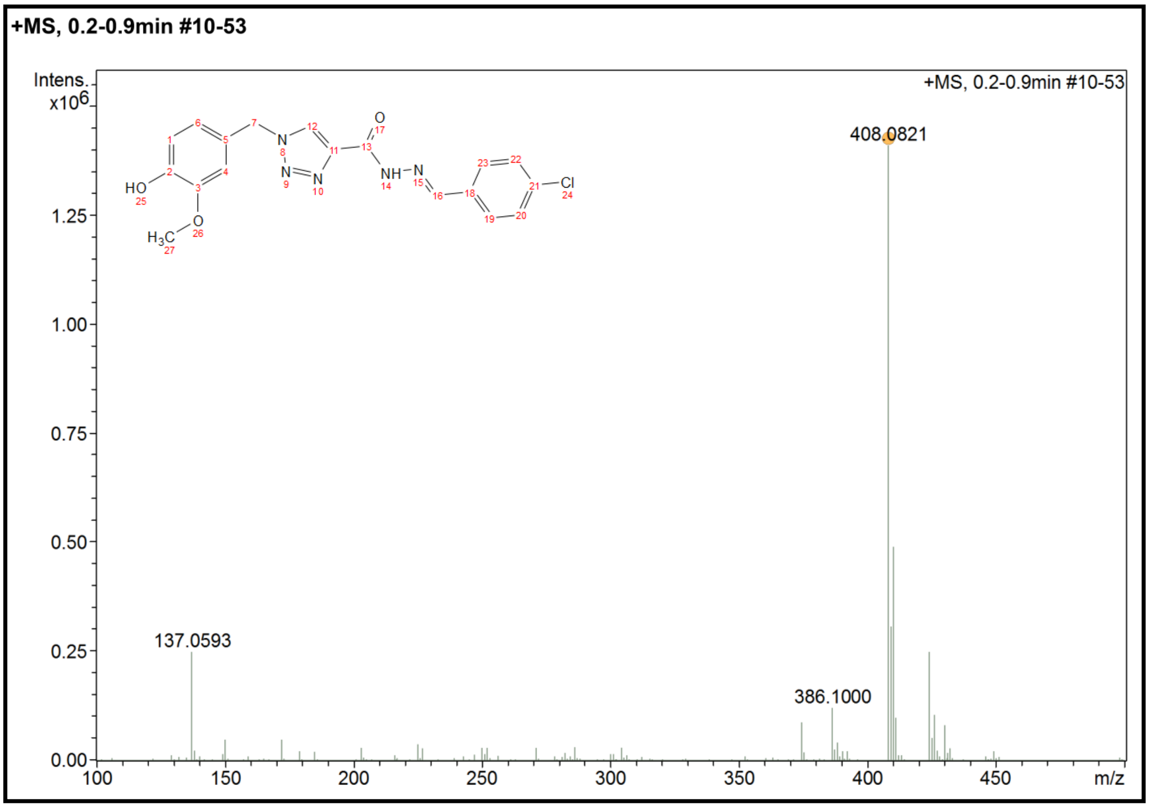

Figure S 23. HR-MS spectra of(E)-N'-(4-chlorobenzylidene)-1-(4-hydroxy-3-methoxybenzyl)-1H-1,2,3-triazole-4-carbohydrazide (**3h**).

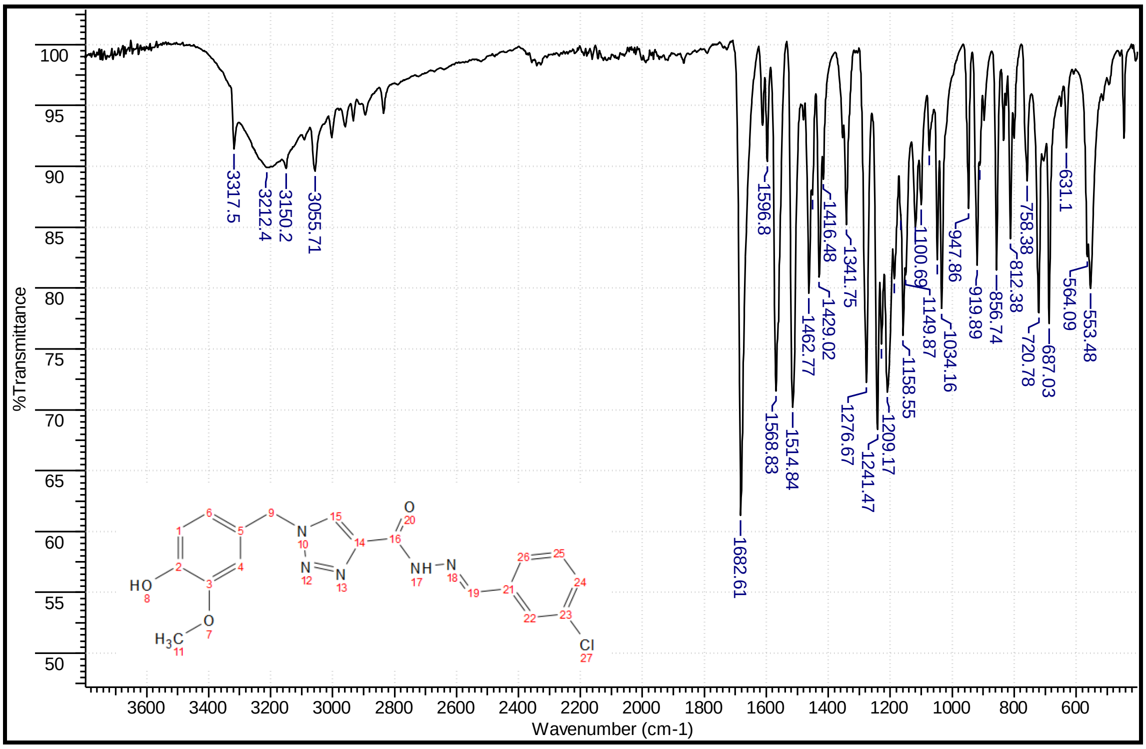

Figure S 24. Absorption spectra in the infrared region of (E)-N'-(3-chlorobenzylidene)-1-(4-hydroxy-3-methoxybenzyl)-1H-1,2,3-triazole-4-carbohydrazide (**3i**).

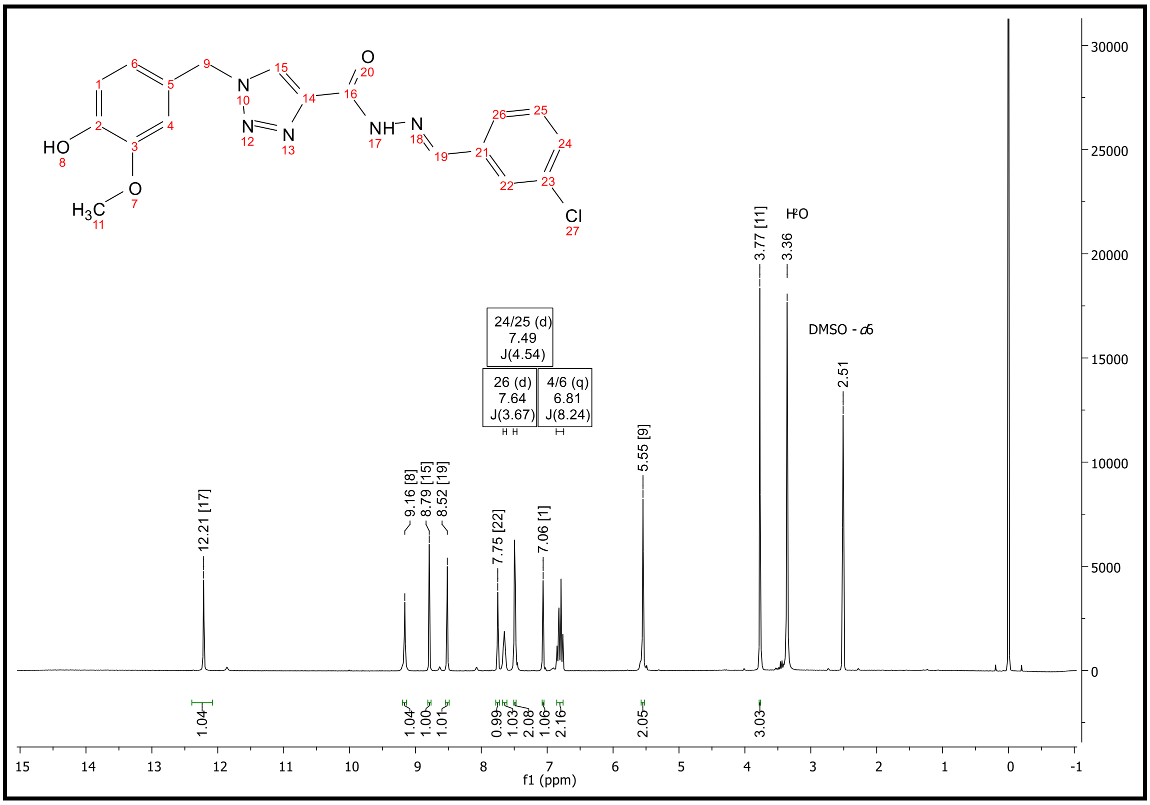

Figure S 25. 1H NMR spectra of (E)-N'-(3-chlorobenzylidene)-1-(4-hydroxy-3-methoxybenzyl)-1H-1,2,3-triazole-4-carbohydrazide (**3i**).

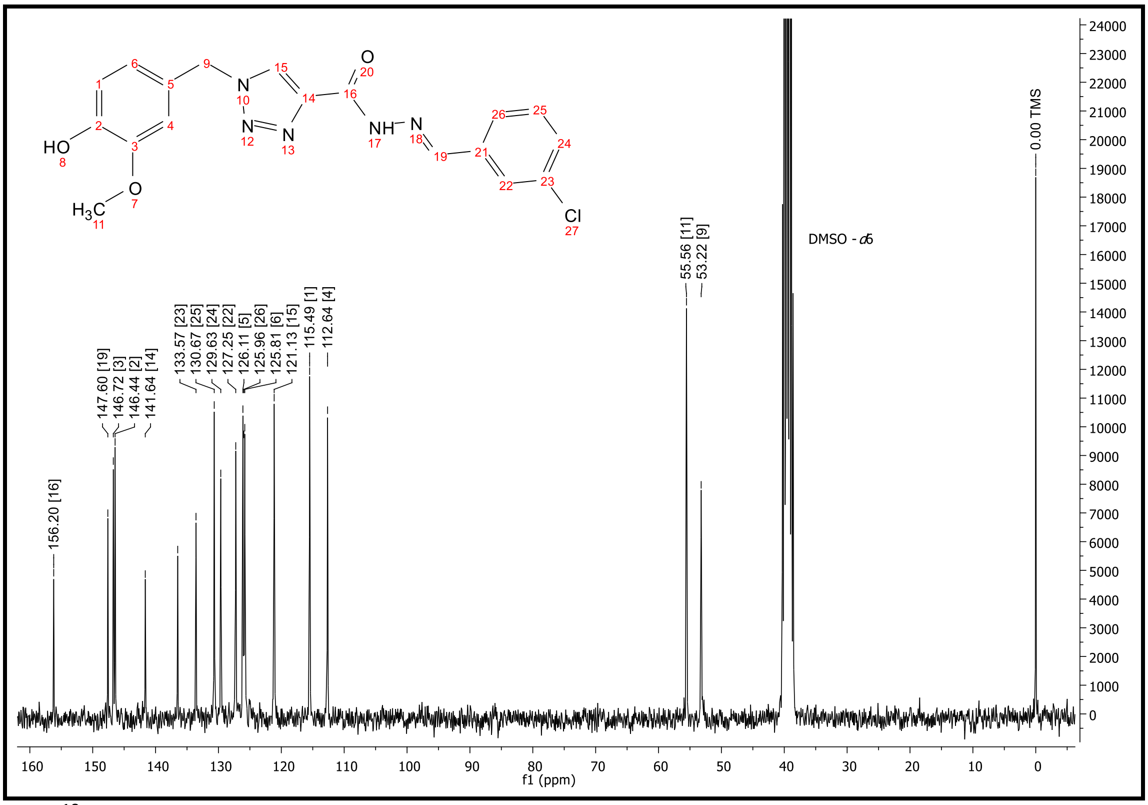

Figure S 26. ^{13}C NMR spectra of (E)-N'-(3-chlorobenzylidene)-1-(4-hydroxy-3-methoxybenzyl)-1H-1,2,3-triazole-4-carbohydrazide (**3i**).

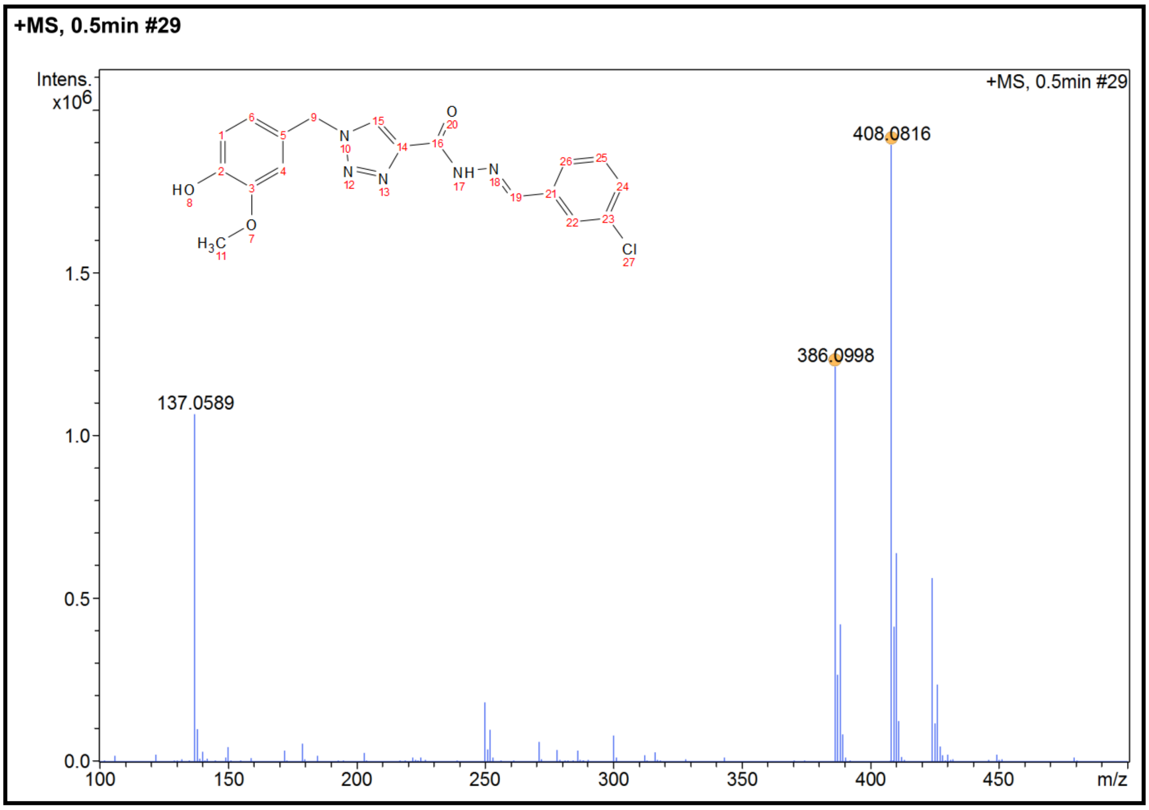

Figure S 27. HR-MS spectra of (E)-N'-(3-chlorobenzylidene)-1-(4-hydroxy-3-methoxybenzyl)-1H-1,2,3-triazole-4-carbohydrazide (**3i**).

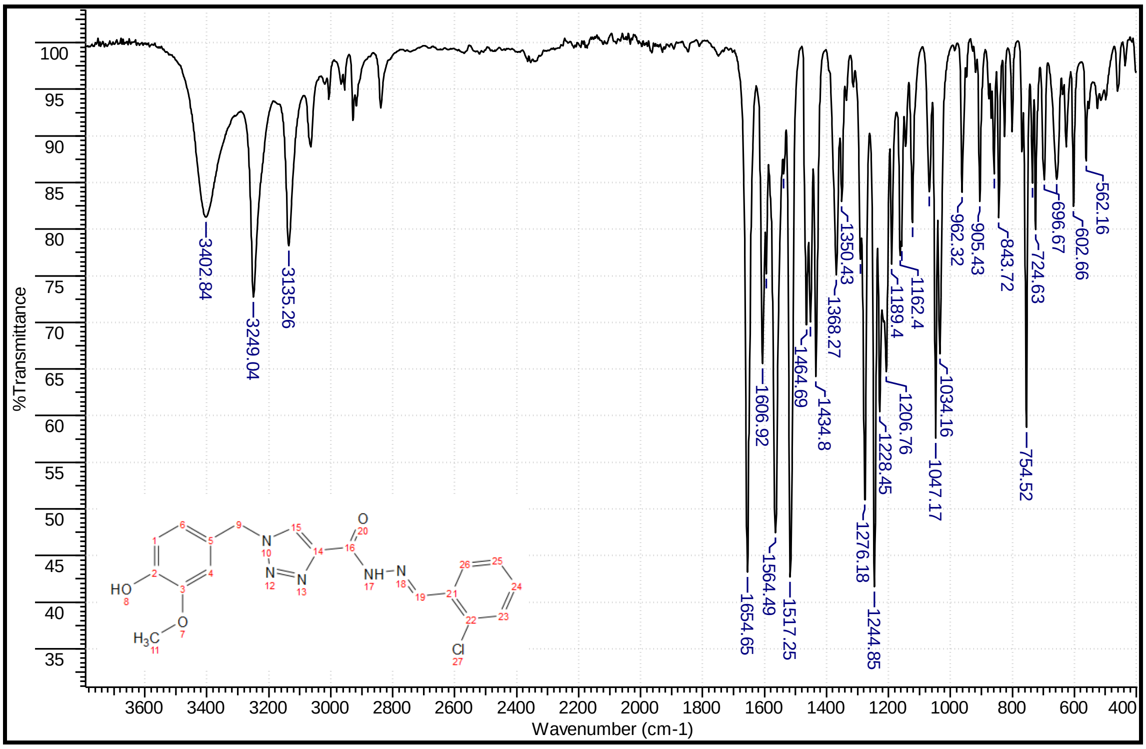

Figure S 28. Absorption spectra in the infrared region of (E)-N'-(2-chlorobenzylidene)-1-(4-hydroxy-3-methoxybenzyl)-1H-1,2,3-triazole-4-carbohydrazide (**3j**).

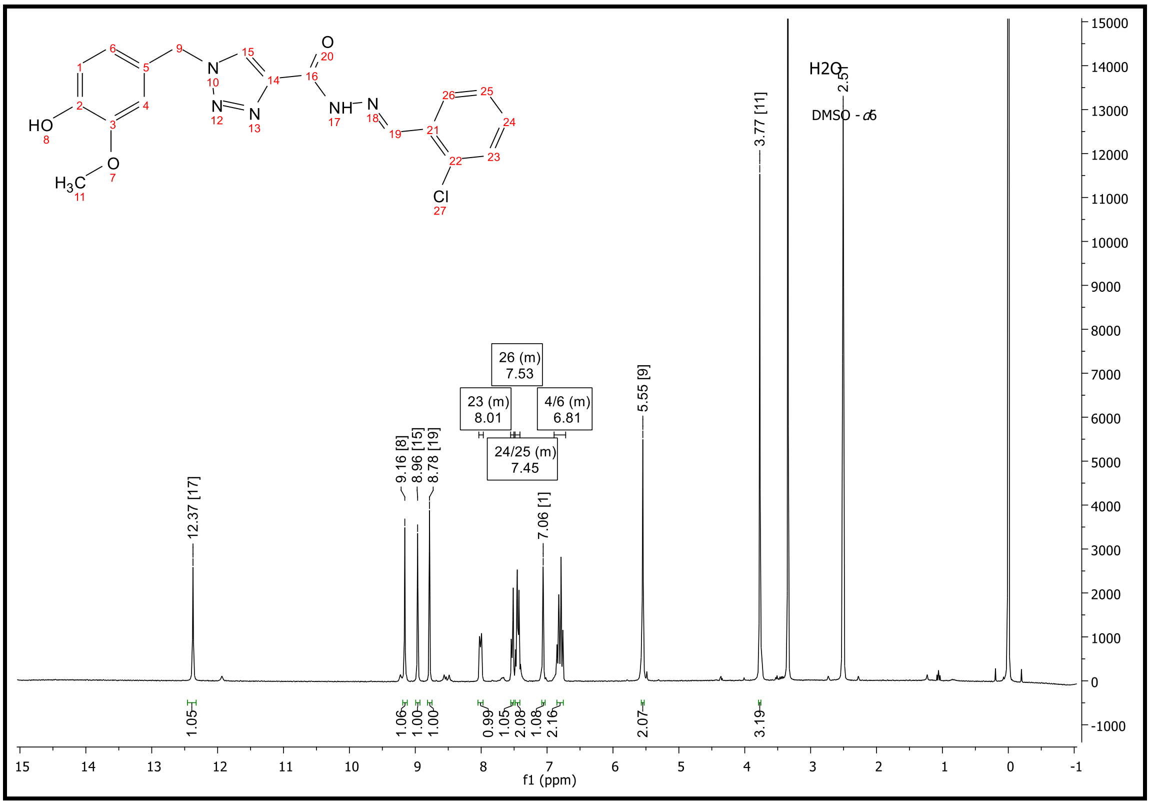

Figure S 29. 1H NMR spectra of (E)-N'-(2-chlorobenzylidene)-1-(4-hydroxy-3-methoxybenzyl)-1H-1,2,3-triazole-4-carbohydrazide (**3j**).

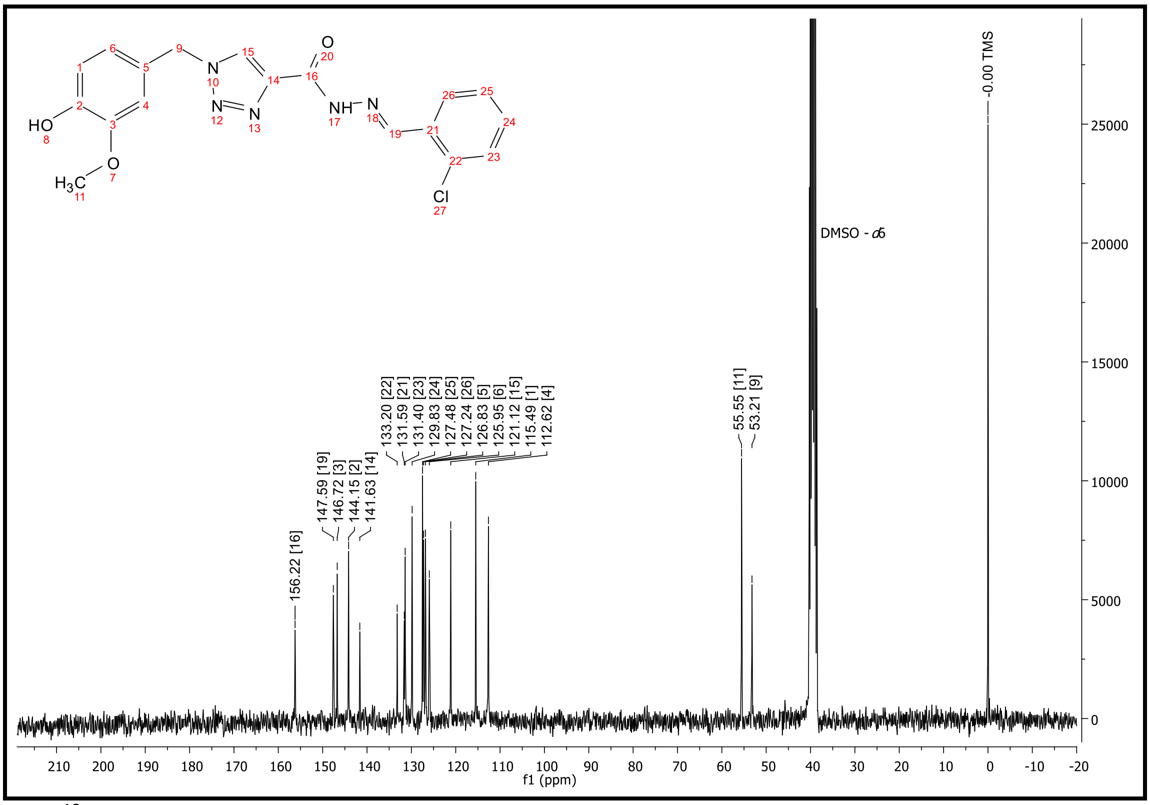

Figure S 30. ^{13}C NMR spectra of (E)-N'-(2-chlorobenzylidene)-1-(4-hydroxy-3-methoxybenzyl)-1H-1,2,3-triazole-4-carbohydrazide (**3j**).

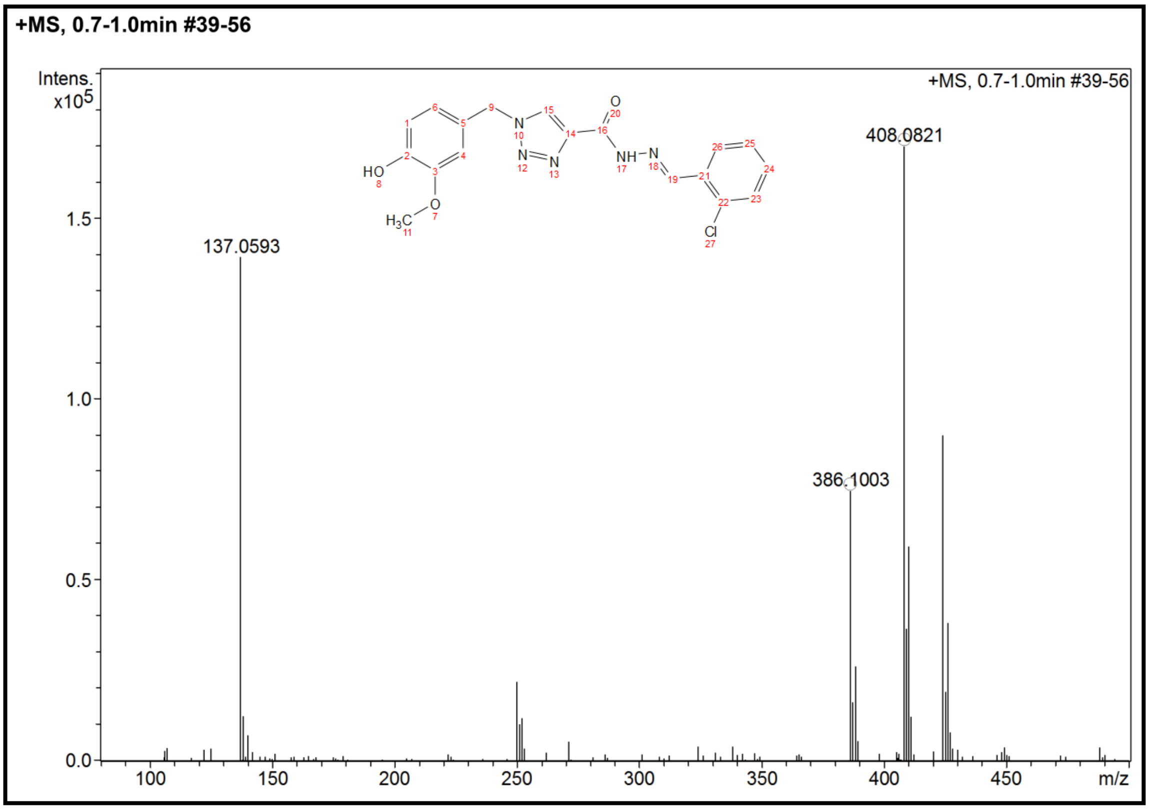

Figure S 31. HR-MS spectra of (E)-N'-(2-chlorobenzylidene)-1-(4-hydroxy-3-methoxybenzyl)-1H-1,2,3-triazole-4-carbohydrazide (**3j**).

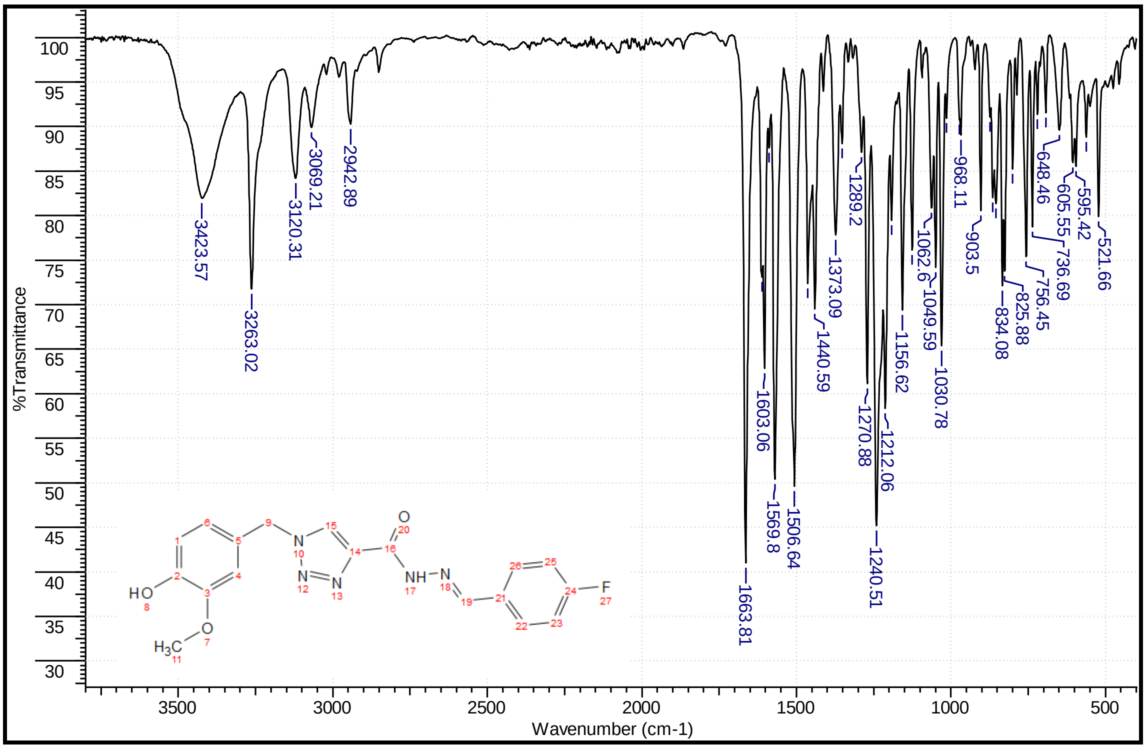

Figure S 32. Absorption spectra in the infrared region of (E)-N'-(4-fluorobenzylidene)-1-(4-hydroxy-3-methoxybenzyl)-1H-1,2,3-triazole-4-carbohydrazide (**3k**).

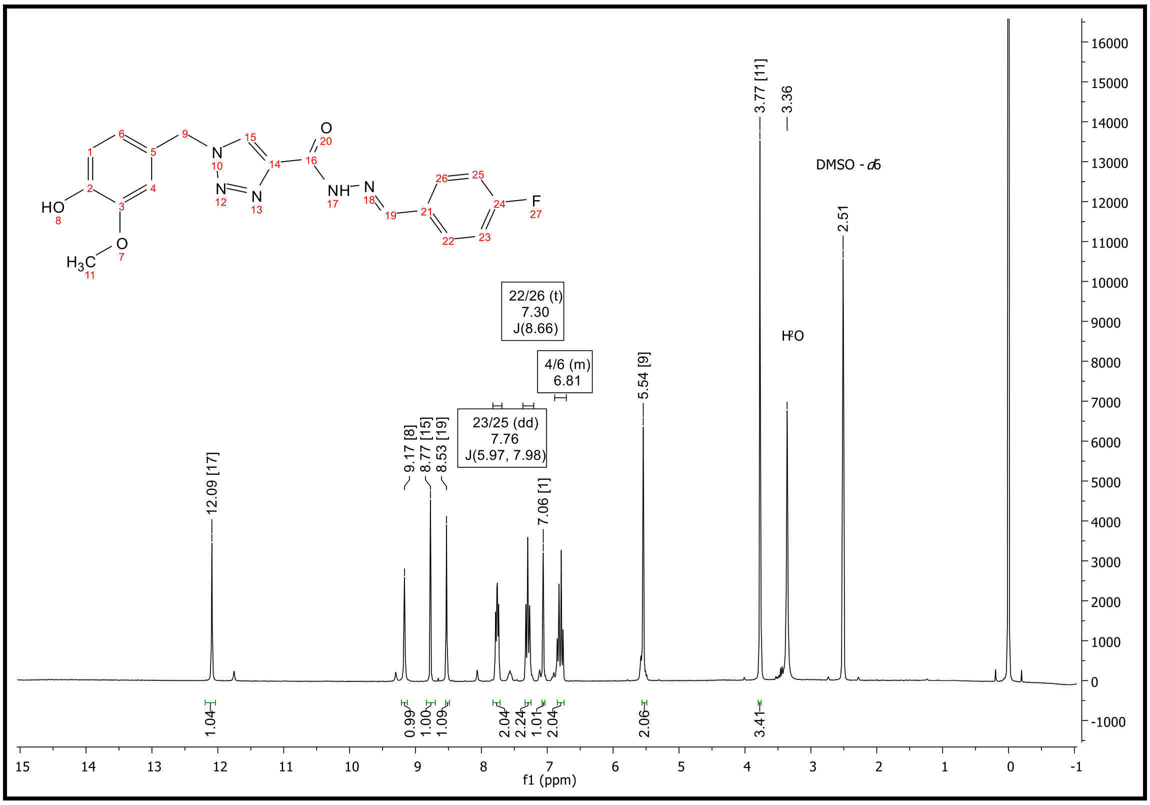

Figure S 33. 1H NMR spectra of (E)-N'-(4-fluorobenzylidene)-1-(4-hydroxy-3-methoxybenzyl)-1H-1,2,3-triazole-4-carbohydrazide (**3k**).

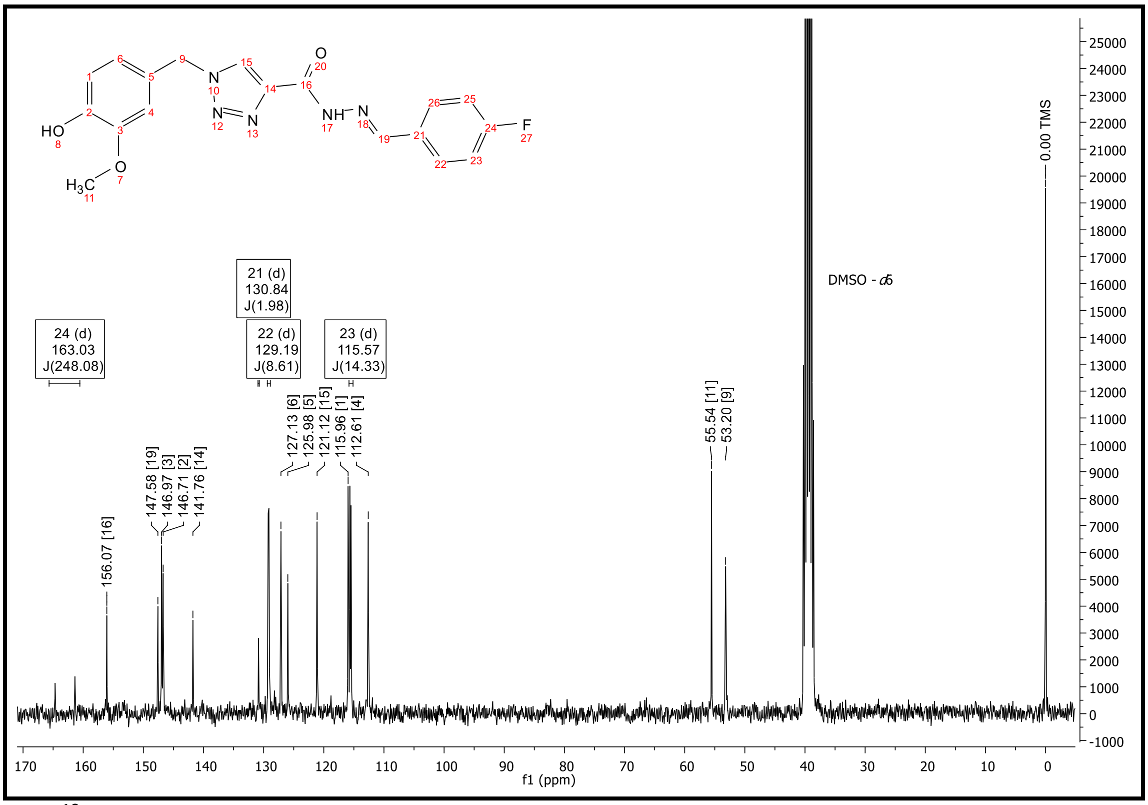

Figure S 34. ^{13}C NMR spectra of (E)-N'-(4-fluorobenzylidene)-1-(4-hydroxy-3-methoxybenzyl)-1H-1,2,3-triazole-4-carbohydrazide (**3k**).

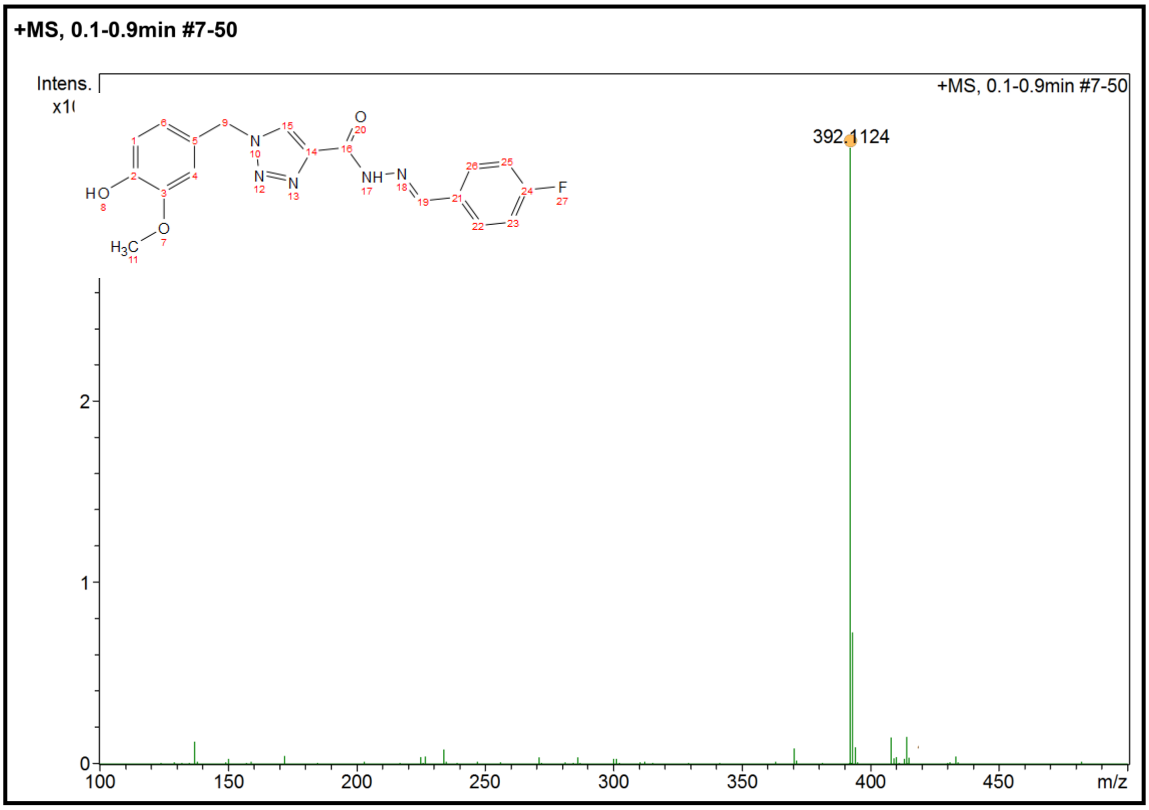

Figure S 35. HR-MS spectra of (E)-N'-(4-fluorobenzylidene)-1-(4-hydroxy-3-methoxybenzyl)-1H-1,2,3-triazole-4-carbohydrazide (**3k**).

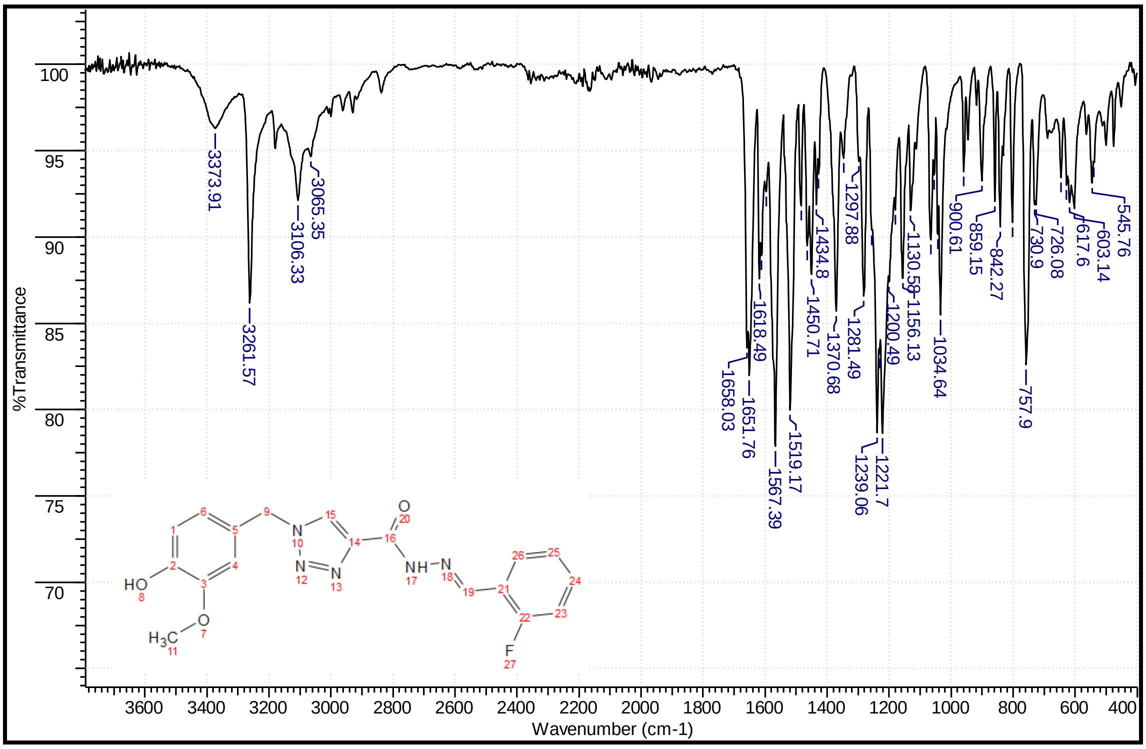

Figure S 36. Absorption spectra in the infrared region of (E)-N'-(2-fluorobenzylidene)-1-(4-hydroxy-3-methoxybenzyl)-1H-1,2,3-triazole-4-carbohydrazide (**3l**).

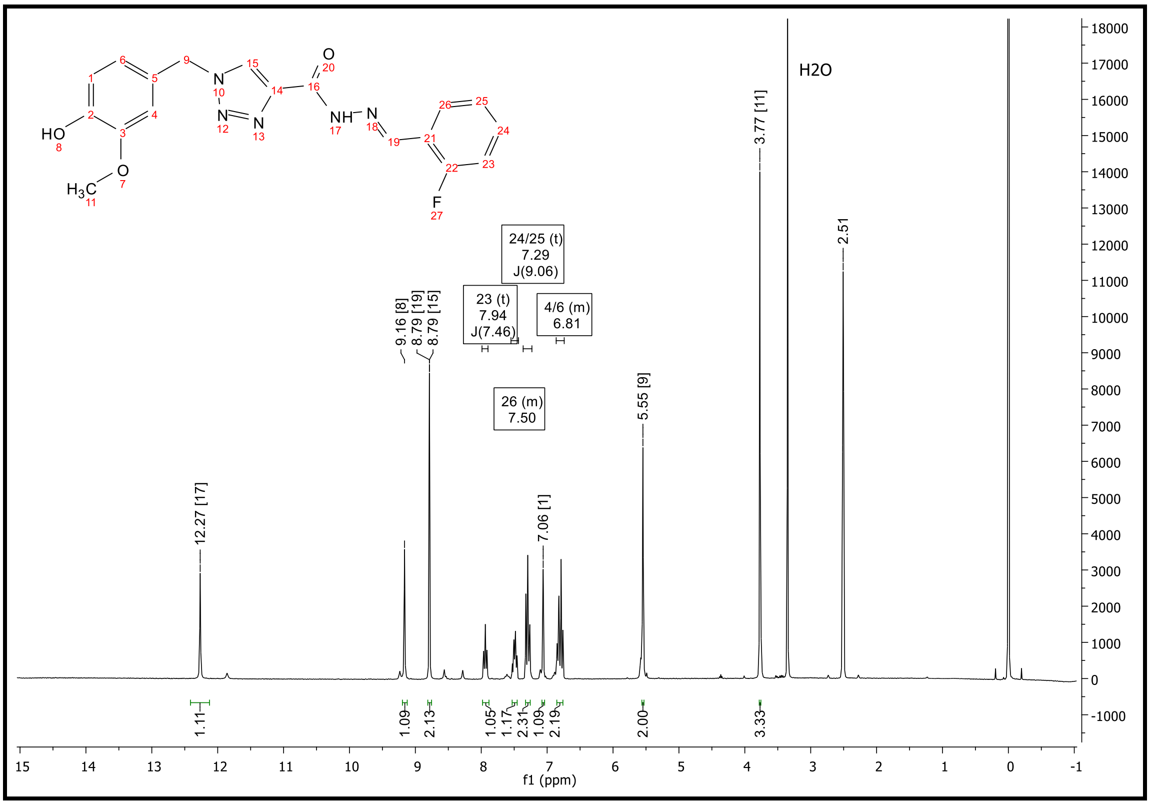

Figure S 37. 1H NMR spectra of (E)-N'-(2-fluorobenzylidene)-1-(4-hydroxy-3-methoxybenzyl)-1H-1,2,3-triazole-4-carbohydrazide (**3l**).

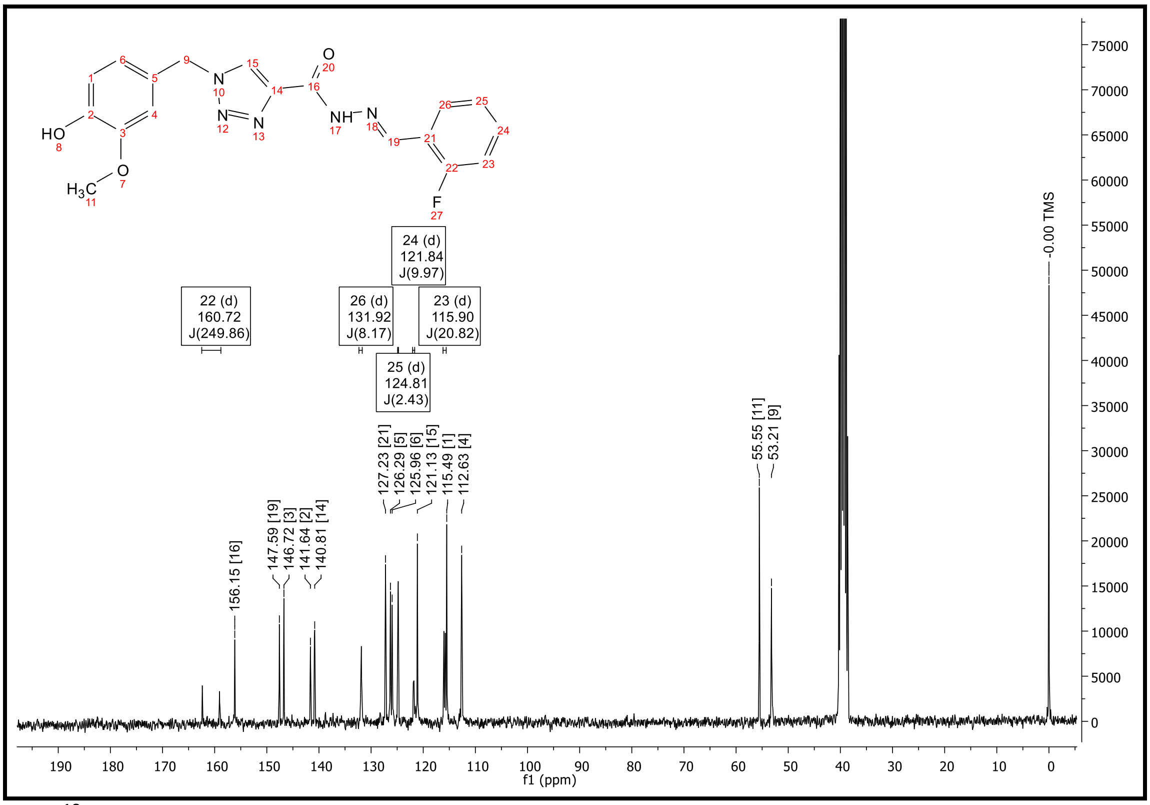

Figure S 38. ^{13}C NMR spectra of (E)-N'-(2-fluorobenzylidene)-1-(4-hydroxy-3-methoxybenzyl)-1H-1,2,3-triazole-4-carbohydrazide (**3l**).

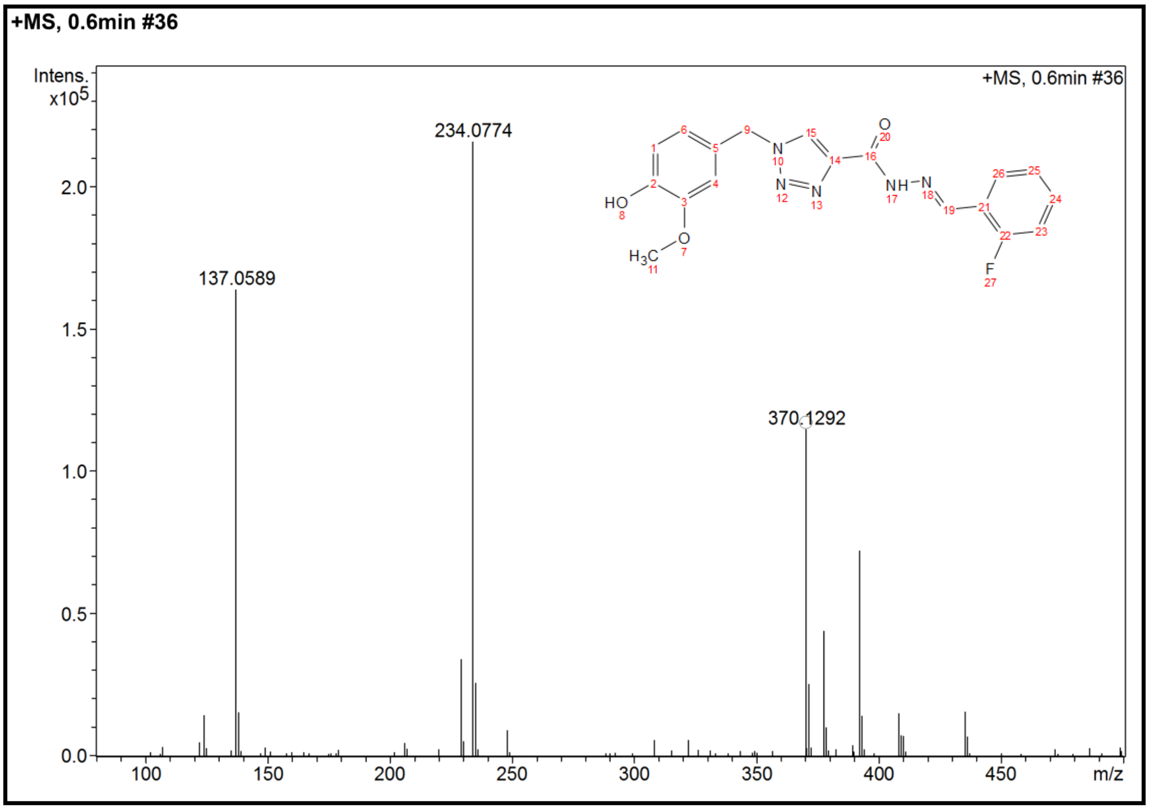

Figure S 39. HR-MS spectra of (E)-N'-(2-fluorobenzylidene)-1-(4-hydroxy-3-methoxybenzyl)-1H-1,2,3-triazole-4-carbohydrazide (**3l**).

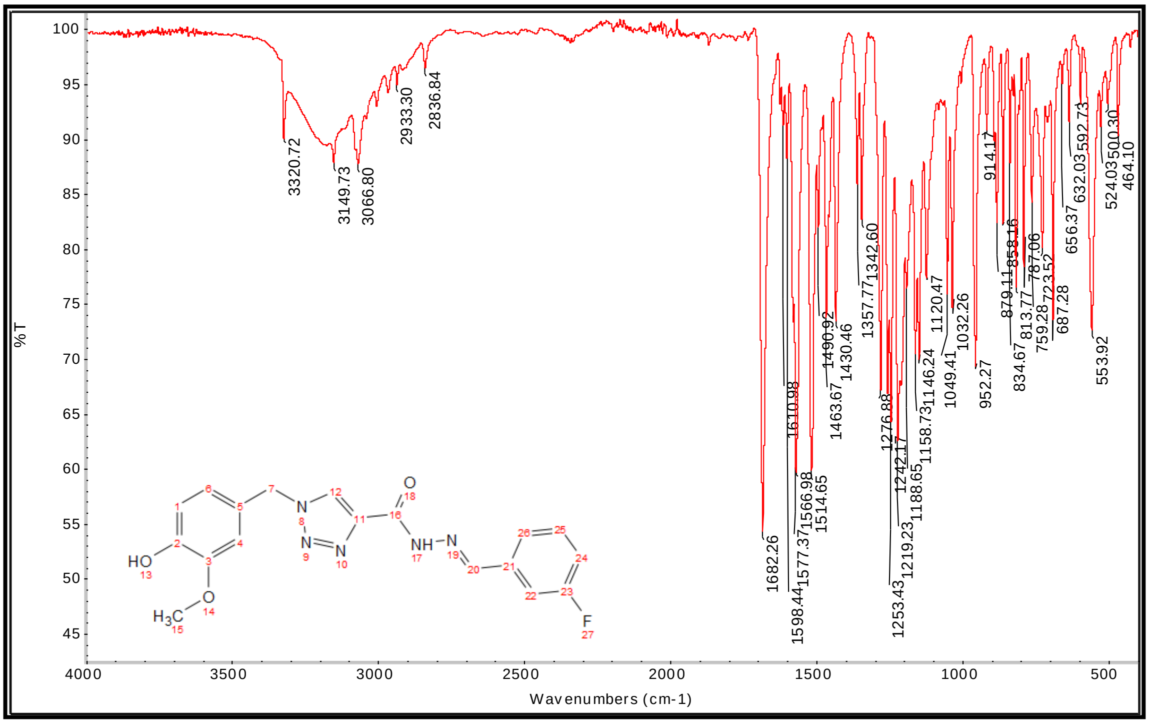

Figure S 40. Absorption spectra in the infrared region of (E)-N'-(3-fluorobenzylidene)-1-(4-hydroxy-3-methoxybenzyl)-1H-1,2,3-triazole-4-carbohydrazide (**3m**).

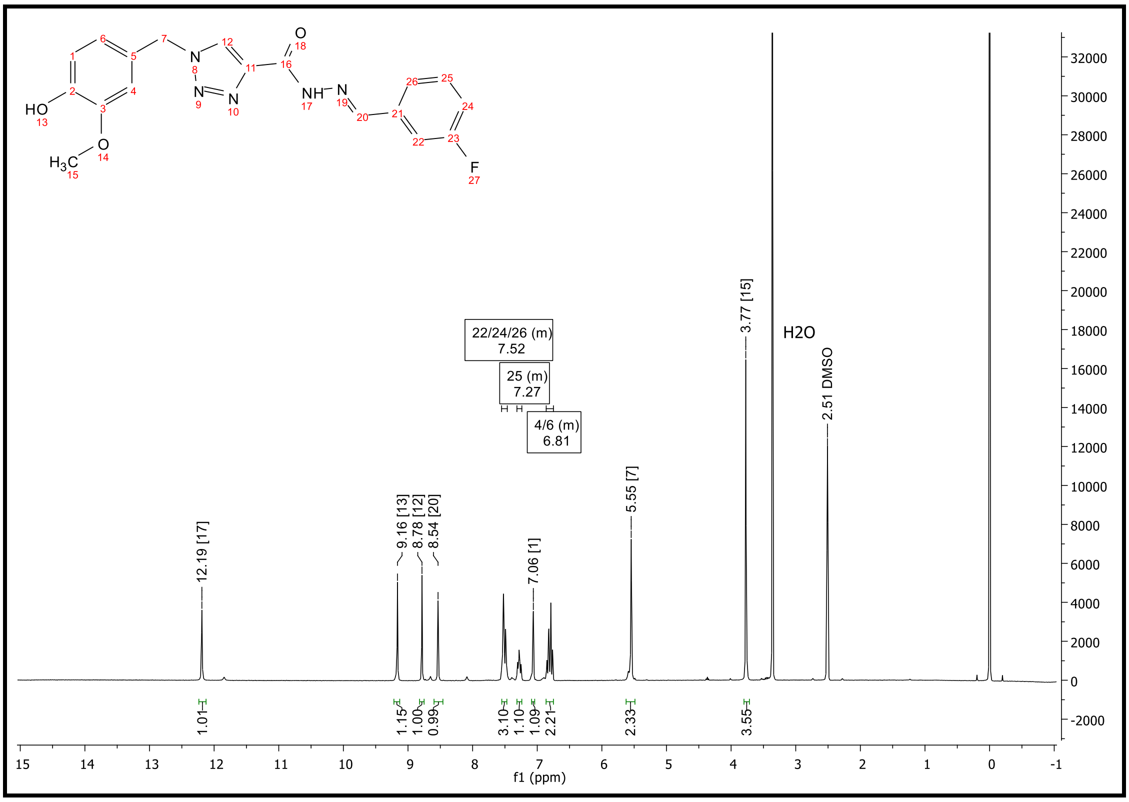

Figure S 41. 1H NMR spectra of (E)-N'-(3-fluorobenzylidene)-1-(4-hydroxy-3-methoxybenzyl)-1H-1,2,3-triazole-4-carbohydrazide (**3m**).

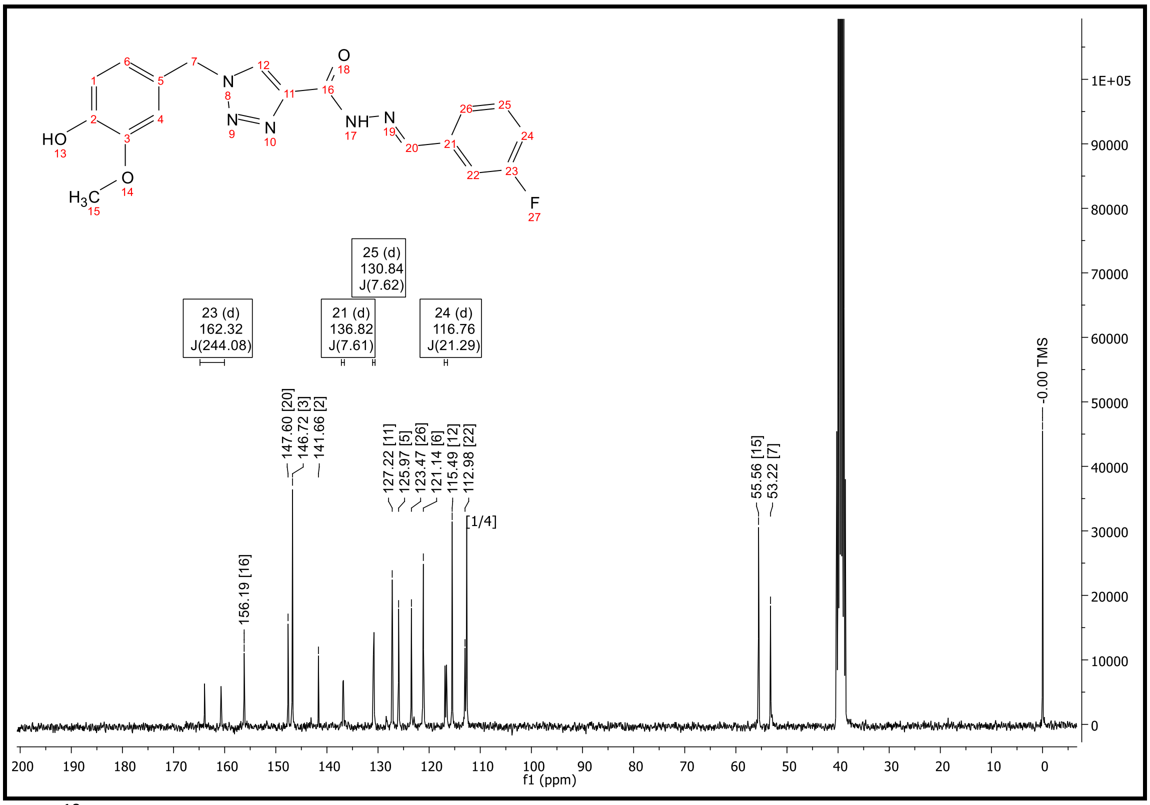

Figure S 42. ^{13}C NMR spectra of (E)-N'-(3-fluorobenzylidene)-1-(4-hydroxy-3-methoxybenzyl)-1H-1,2,3-triazole-4-carbohydrazide (**3m**).

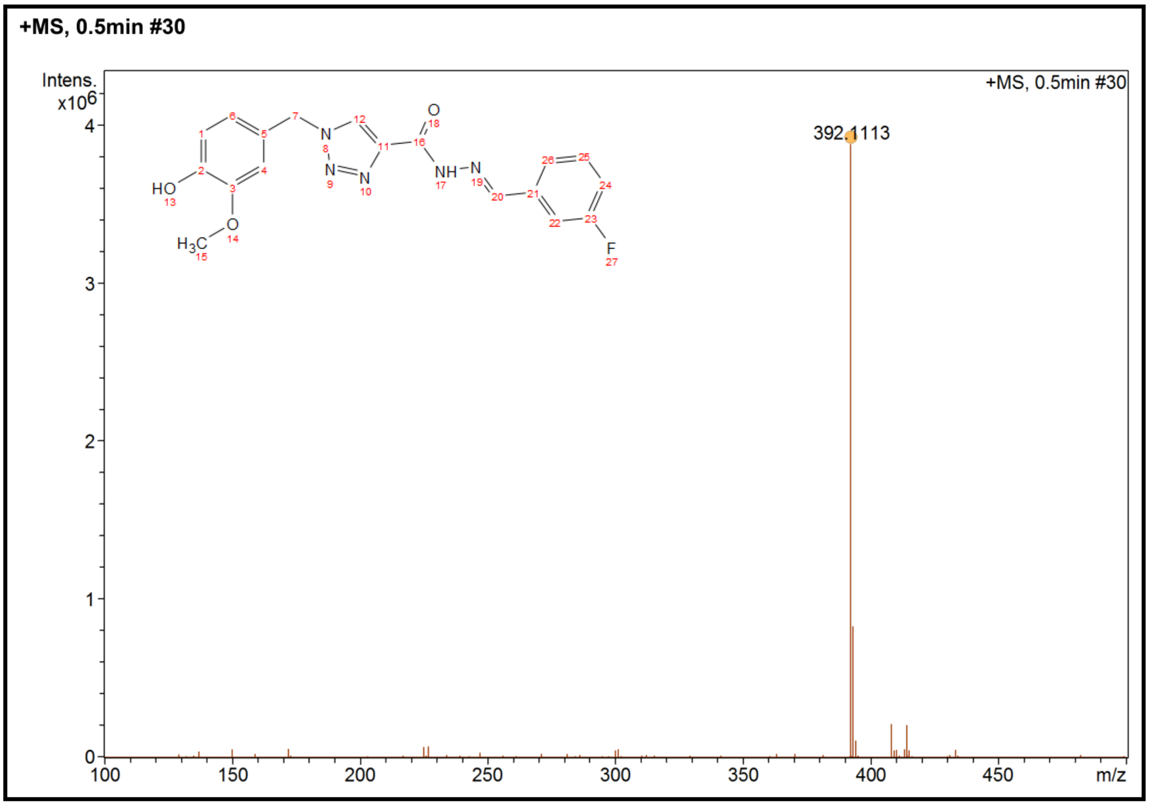

Figure S 43. HR-MS spectra of (E)-N'-(3-fluorobenzylidene)-1-(4-hydroxy-3-methoxybenzyl)-1H-1,2,3-triazole-4-carbohydrazide (**3m**).

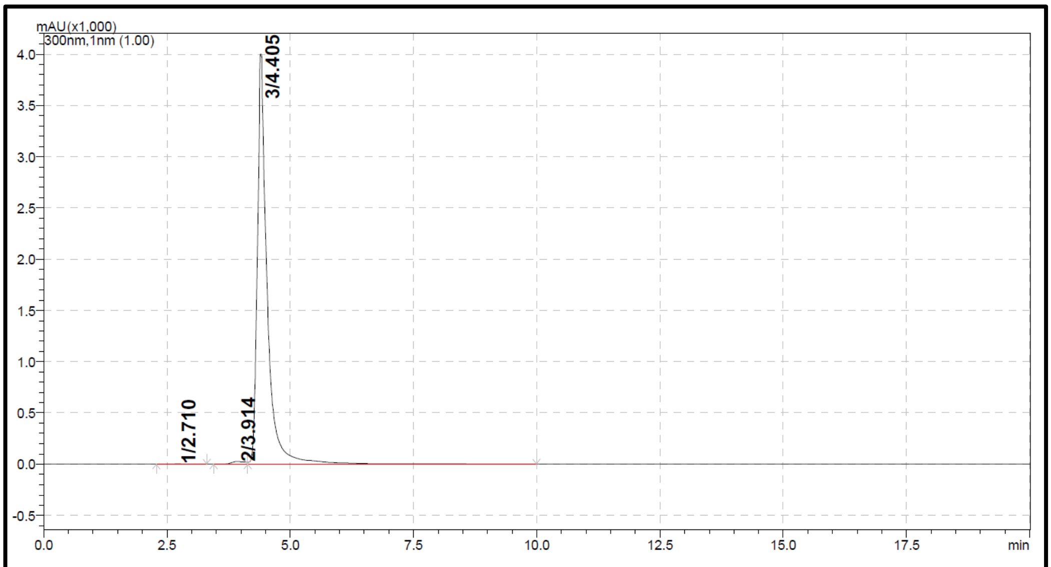

Peak#	Ret. Time	Area	Area%	Peak Start	Peak End
1	2.710	36368	0.0653	2.283	3.307
2	3.914	479805	0.8609	3.445	4.139
3	4.405	55216608	99.0738	4.139	9.995

Figure S 44. Chromatogram of (E)-1-(4-hydroxy-3-methoxybenzyl)-N'-(4-hydroxybenzylidene)-1H-1,2,3-triazole-4-carbohydrazide (**3a**).

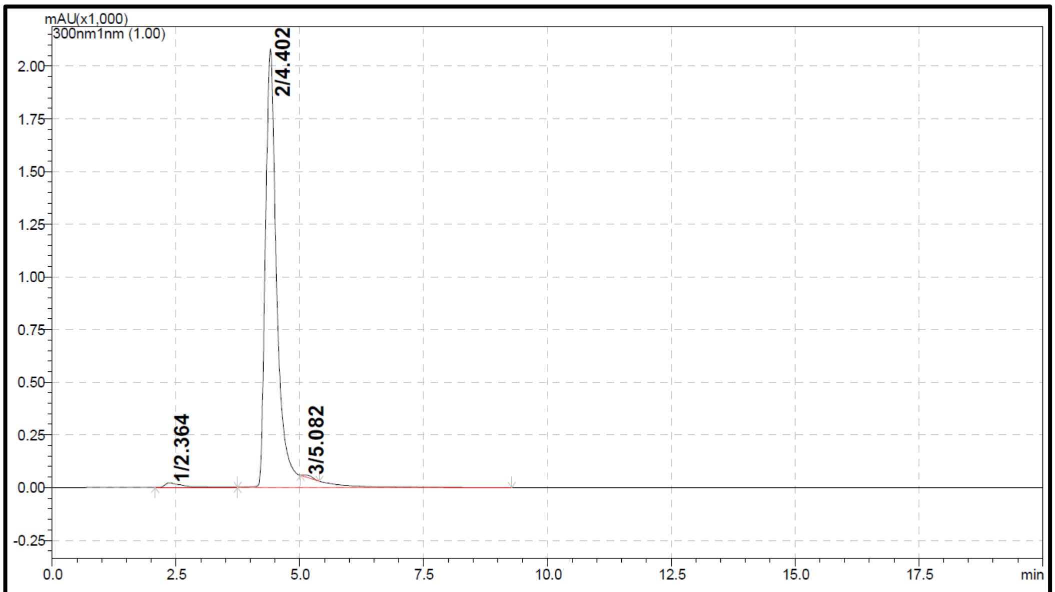

Peak#	Ret. Time	Area	Area%	Peak Start	Peak End
1	2.364	555826	1.5539	2.080	3.744
2	4.402	35086147	98.0904	3.744	9.280
3	5.082	127232	0.3557	5.013	5.397

Figure S 45. Chromatogram of (E)-N'-(3,5-dihydroxybenzylidene)-1-(4-hydroxy-3-methoxybenzyl)-1H-1,2,3-triazole-4-carbohydrazide (**3b**).

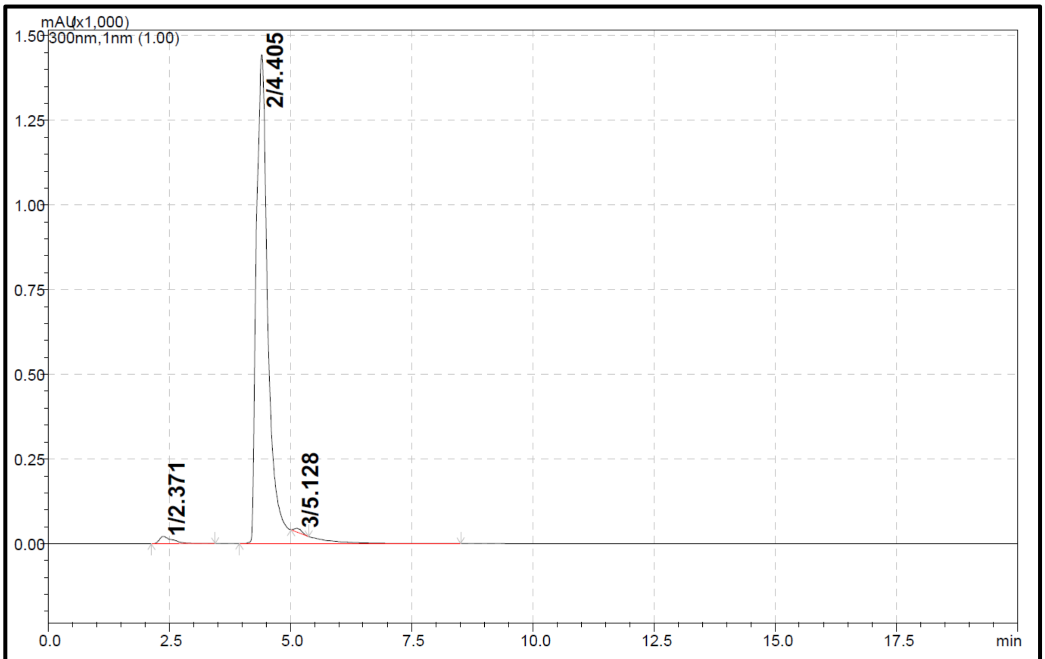

Peak#	Ret. Time	Area	Area%	Peak Start	Peak End
1	2.371	431716	1.6792	2.133	3.445
2	4.405	25159646	97.8591	3.947	8.512
3	5.128	118708	0.4617	5.013	5.376

Figure S 46. Chromatogram of (E)-N'-(3,5-dimethoxybenzylidene)-1-(4-hydroxy-3-methoxybenzyl)-1H-1,2,3-triazole-4-carbohydrazide (**3d**).

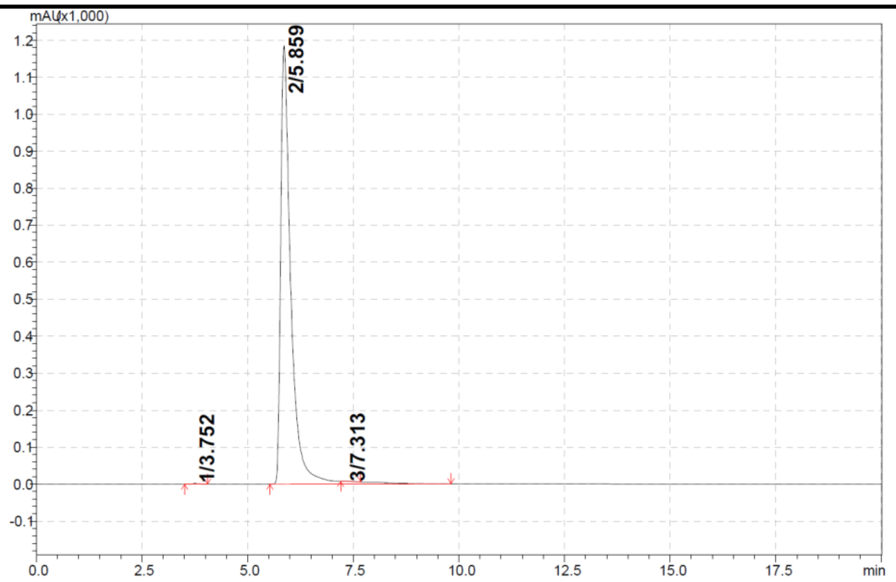

Peak#	Ret. Time	Area	Area%	Peak Start	Peak End
1	3.752	16837	0.0839	3.509	4.053
2	5.859	20031710	99.8639	5.525	9.813
3	7.313	10465	0.0522	7.200	7.680

Figure S 47. Chromatogram of (E)-1-(4-hydroxy-3-methoxybenzyl)-N'-(4-hydroxy-3-methoxybenzylidene)-1H-1,2,3-triazole-4-carbohydrazide (**3e**).

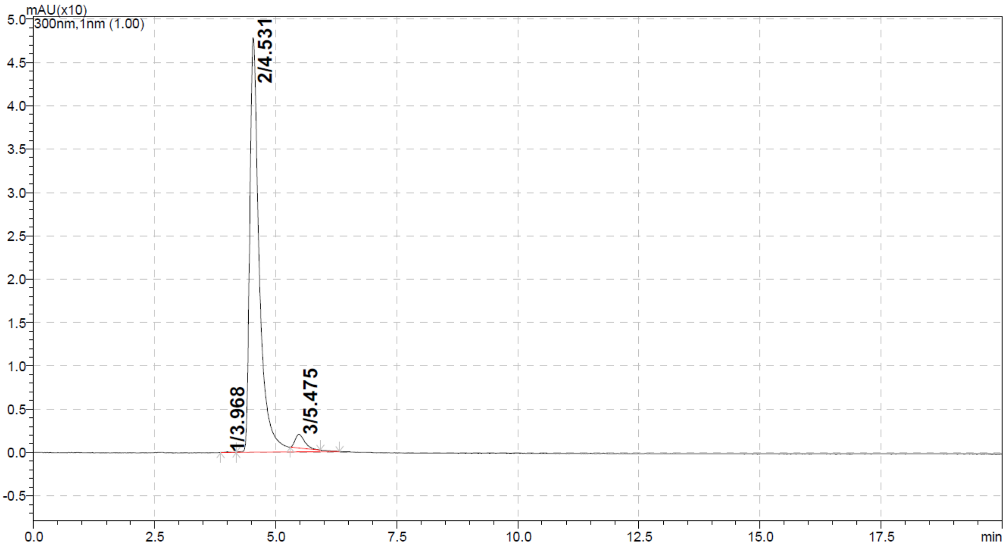

Peak#	Ret. Time	Area	Area%	Peak Start	Peak End
1	3.968	1025	0.1478	3.861	4.181
2	4.531	671143	96.7855	4.181	6.315
3	5.475	21265	3.0667	5.301	5.920

Figure S 48. Chromatogram of (E)-N'-benzylidene-1-(4-hydroxy-3-methoxybenzyl)-1H-1,2,3-triazole-4-carbohydrazide (**3f**).

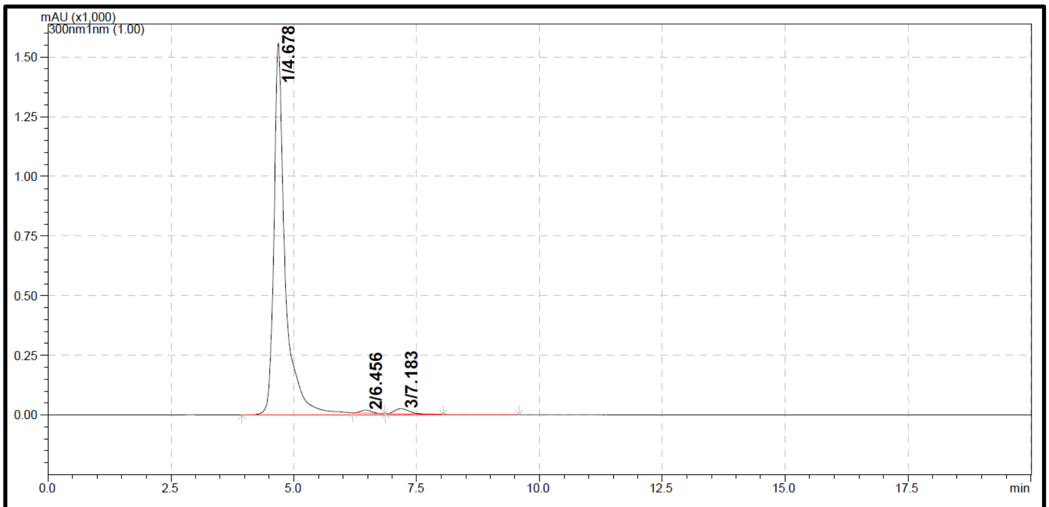

Peak#	Ret. Time	Area	Area%	Peak Start	Peak End
1	4.678	25622782	97.4183	3.947	9.579
2	6.456	209393	0.7961	6.208	6.848
3	7.183	469641	1.7856	6.869	8.043

Figure S 49. Chromatogram of (E)-1-(4-hydroxy-3-methoxybenzyl)-N'-(4-(piperidin-1-yl)benzylidene)-1H-1,2,3-triazole-4-carbohydrazide (**3g**).

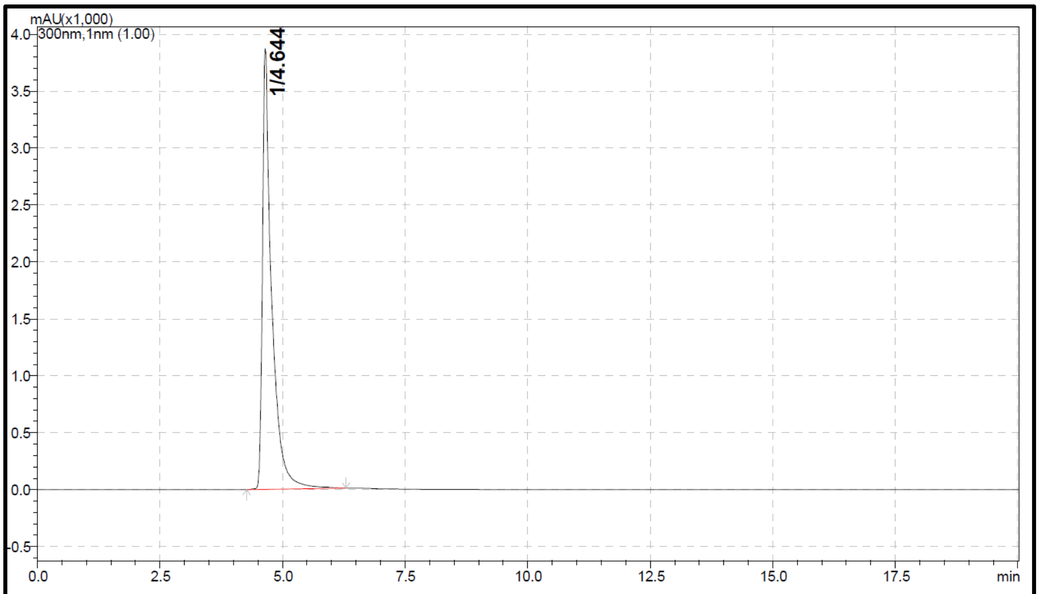

Peak#	Ret. Time	Area	Area%	Peak Start	Peak End
1	4.644	50770984	100.0000	4.267	6.293

Figure S 50. Chromatogram of (E)-N'-(4-chlorobenzylidene)-1-(4-hydroxy-3-methoxybenzyl)-1H-1,2,3-triazole-4-carbohydrazide (**3h**).

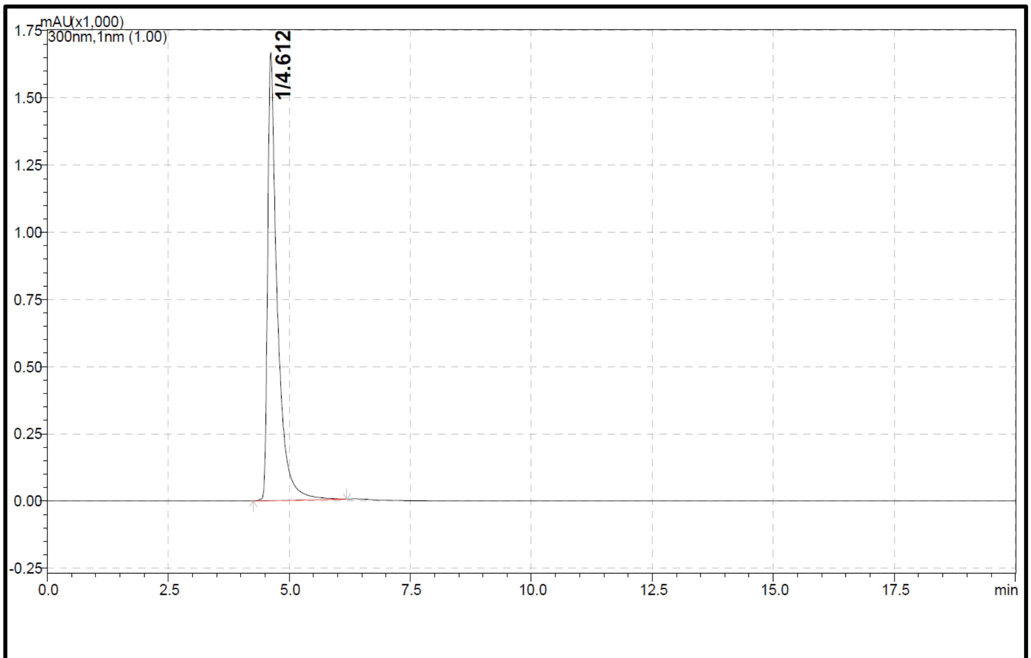

Peak#	Ret. Time	Area	Area%	Peak Start	Peak End
1	4.612	23177742	100.0000	4.256	6.187

Figure S 51. Chromatogram of (E)-N'-(3-chlorobenzylidene)-1-(4-hydroxy-3-methoxybenzyl)-1H-1,2,3-triazole-4-carbohydrazide (**3i**).

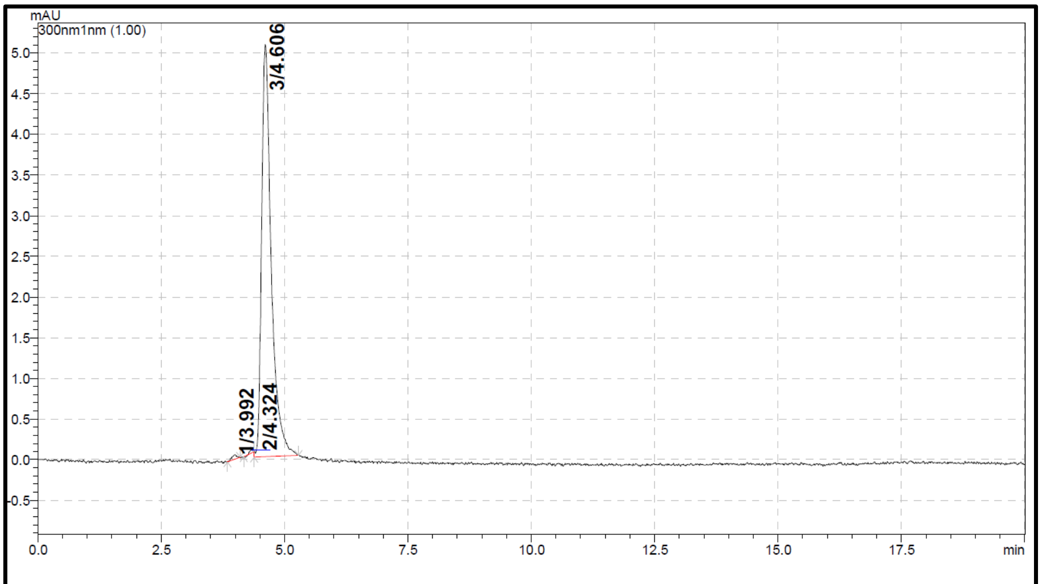

Peak#	Ret. Time	Area	Area%	Peak Start	Peak End
1	3.992	388	0.5614	3.840	4.096
2	4.324	143	0.2063	4.171	4.384
3	4.606	68655	99.2323	4.384	5.280

Figure S 52. Chromatogram of (E)-N'-(2-chlorobenzylidene)-1-(4-hydroxy-3-methoxybenzyl)-1H-1,2,3-triazole-4-carbohydrazide (**3j**).

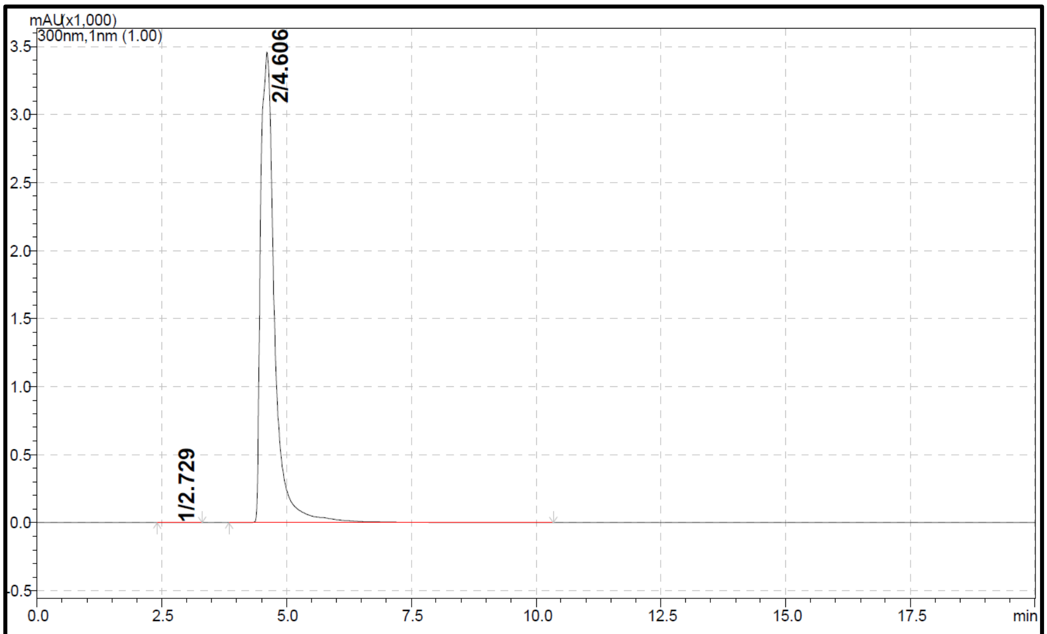

Peak#	Ret. Time	Area	Area%	Peak Start	Peak End
1	2.729	26323	0.0390	2.411	3.307
2	4.606	67387652	99.9610	3.851	10.347

Figure S 53. Chromatogram of (E)-N'-(4-fluorobenzylidene)-1-(4-hydroxy-3-methoxybenzyl)-1H-1,2,3-triazole-4-carbohydrazide (**3k**).

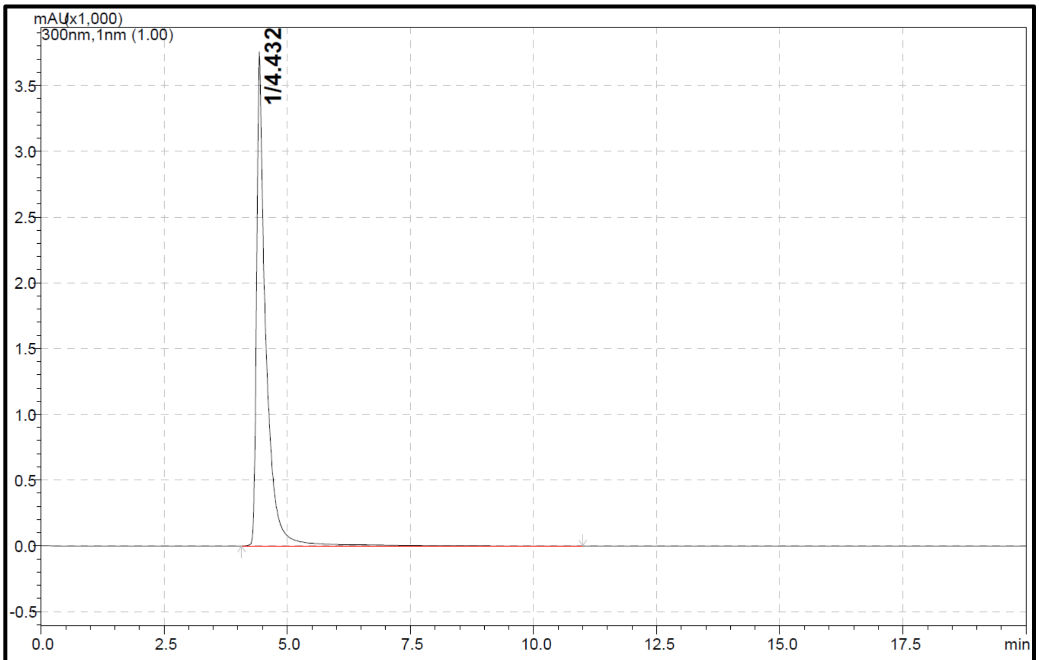

Peak#	Ret. Time	Area	Area%	Peak Start	Peak End
1	4.432	49169491	100.0000	4.075	10.997

Figure S 54. Chromatogram of (E)-N'-(2-fluorobenzylidene)-1-(4-hydroxy-3-methoxybenzyl)-1H-1,2,3-triazole-4-carbohydrazide (**3l**).

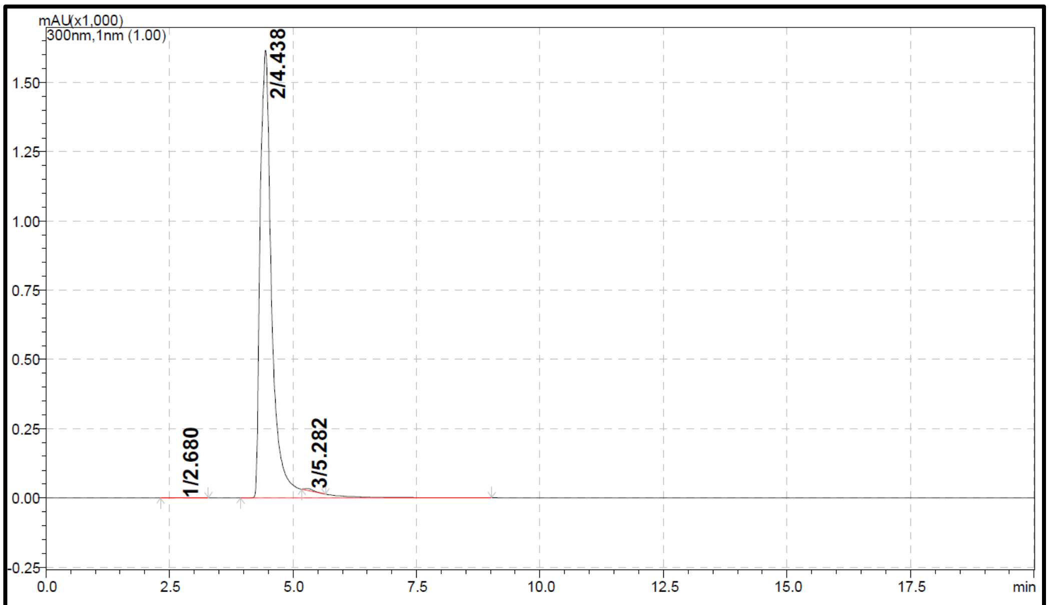

Peak#	Ret. Time	Area	Area%	Peak Start	Peak End
1	2.680	14641	0.0537	2.315	3.285
2	4.438	27191892	99.6527	3.936	9.024
3	5.282	80134	0.2937	5.173	5.653

Figure S 55. Chromatogram of (E)-N'-(3-fluorobenzylidene)-1-(4-hydroxy-3-methoxybenzyl)-1H-1,2,3-triazole-4-carbohydrazide (**3m**).
